# The longevity-associated BPIFB4 gene supports cardiac function and vascularization in ageing cardiomyopathy

**DOI:** 10.1093/cvr/cvad008

**Published:** 2023-01-13

**Authors:** Monica Cattaneo, Antonio P Beltrami, Anita C Thomas, Gaia Spinetti, Valeria Vincenza Alvino, Elisa Avolio, Claudia Veneziano, Irene Giulia Rolle, Sandro Sponga, Elena Sangalli, Anna Maciag, Fabrizio Dal Piaz, Carmine Vecchione, Aishah Alenezi, Stephen Paisey, Annibale A Puca, Paolo Madeddu

**Affiliations:** Cardiovascular Department, IRCCS MultiMedica, Via G. Fantoli, 16/15, 20138 Milan, Italy; Department of Medicine, University of Udine, Academic Hospital of Udine, ASUFC, Piazzale Santa Maria della Misercordia 15, 33100 Udine, Italy; Translational Health Sciences, Bristol Medical School, University of Bristol, Upper Maudlin St, Bristol BS2 8HW, UK; Cardiovascular Department, IRCCS MultiMedica, Via G. Fantoli, 16/15, 20138 Milan, Italy; Translational Health Sciences, Bristol Medical School, University of Bristol, Upper Maudlin St, Bristol BS2 8HW, UK; Translational Health Sciences, Bristol Medical School, University of Bristol, Upper Maudlin St, Bristol BS2 8HW, UK; Department of Medicine, University of Udine, Academic Hospital of Udine, ASUFC, Piazzale Santa Maria della Misercordia 15, 33100 Udine, Italy; Department of Medicine, University of Udine, Academic Hospital of Udine, ASUFC, Piazzale Santa Maria della Misercordia 15, 33100 Udine, Italy; Department of Medicine, University of Udine, Academic Hospital of Udine, ASUFC, Piazzale Santa Maria della Misercordia 15, 33100 Udine, Italy; Cardiovascular Department, IRCCS MultiMedica, Via G. Fantoli, 16/15, 20138 Milan, Italy; Cardiovascular Department, IRCCS MultiMedica, Via G. Fantoli, 16/15, 20138 Milan, Italy; Department of Medicine, Surgery and Dentistry, University of Salerno, Via Salvator Allende 1, 84081 Salerno, Italy; Department of Medicine, Surgery and Dentistry, University of Salerno, Via Salvator Allende 1, 84081 Salerno, Italy; Department of Vascular Physiopathology, IRCCS Neuromed, Via Atinense, 18, 86077 Pozzilli IS, Italy; Wales Research & Diagnostic Positron Emission Tomography Imaging centre (PETIC) School of Medicine, Heath Park, Cardiff University, CF14 4XN Cardiff, UK; Wales Research & Diagnostic Positron Emission Tomography Imaging centre (PETIC) School of Medicine, Heath Park, Cardiff University, CF14 4XN Cardiff, UK; Cardiovascular Department, IRCCS MultiMedica, Via G. Fantoli, 16/15, 20138 Milan, Italy; Department of Medicine, Surgery and Dentistry, University of Salerno, Via Salvator Allende 1, 84081 Salerno, Italy; Translational Health Sciences, Bristol Medical School, University of Bristol, Upper Maudlin St, Bristol BS2 8HW, UK

**Keywords:** Ageing, Angiogenesis, Myocardial fibrosis, Nucleolin, Perivascular cells

## Abstract

**Aims:**

The ageing heart naturally incurs a progressive decline in function and perfusion that available treatments cannot halt. However, some exceptional individuals maintain good health until the very late stage of their life due to favourable gene–environment interaction. We have previously shown that carriers of a longevity-associated variant (*LAV*) of the *BPIFB4* gene enjoy prolonged health spans and lesser cardiovascular complications. Moreover, supplementation of *LAV-BPIFB4 via* an adeno-associated viral vector improves cardiovascular performance in limb ischaemia, atherosclerosis, and diabetes models. Here, we asked whether the *LAV-BPIFB4* gene could address the unmet therapeutic need to delay the heart’s spontaneous ageing.

**Methods and results:**

Immunohistological studies showed a remarkable reduction in vessel coverage by pericytes in failing hearts explanted from elderly patients. This defect was attenuated in patients carrying the homozygous *LAV-BPIFB4* genotype. Moreover, pericytes isolated from older hearts showed low levels of BPIFB4, depressed pro-angiogenic activity, and loss of ribosome biogenesis. *LAV-BPIFB4* supplementation restored pericyte function and pericyte-endothelial cell interactions through a mechanism involving the nucleolar protein nucleolin. Conversely, *BPIFB4* silencing in normal pericytes mimed the heart failure pericytes. Finally, gene therapy with *LAV-BPIFB4* prevented cardiac deterioration in middle-aged mice and rescued cardiac function and myocardial perfusion in older mice by improving microvasculature density and pericyte coverage.

**Conclusions:**

We report the success of the LAV-BPIFB4 gene/protein in improving homeostatic processes in the heart’s ageing. These findings open to using LAV-BPIFB4 to reverse the decline of heart performance in older people.

## Graphical Abstract

**Graphical Abstract:**
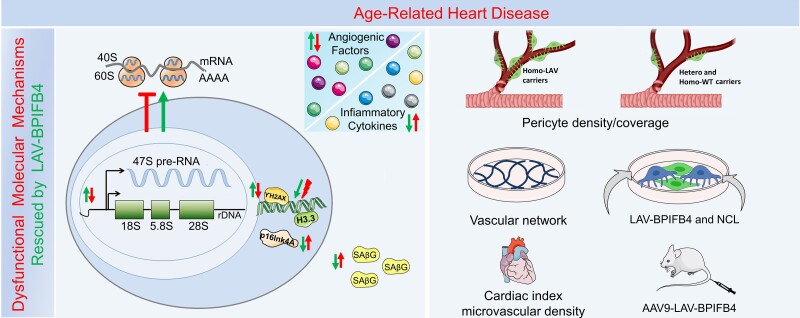
Schematic diagram summarizes dysfunctional key mechanisms and phenotypical features of cardiac ageing that are rescued by LAV-BPIFB4. Cardiac ageing is characterized by abnormal ribosomal biogenesis, DNA damage and senescence, and imbalance of inflammation and angiogenesis (red symbols and arrows). *Left panel: Dysfunctional mechanisms rescued by LAV-BPIFB4 (green arrows).* (i) Ribosomal biogenesis: LAV-BPIFB4 promotes rRNA transcription and ribosomal biogenesis. (ii) Senescence: LAV-BPIFB4 reduces the frequency of the Ki67^neg^ and gH2AX^pos^ antigenic phenotype and senescence markers SAβG, p16lnk4A and H3. (iii) Angiogenesis: LAV-BPIFB4 synergically works with NCL to induce vascular cell network formation. *Right panel: LAV-BPIFB4 in carriers and effects of LAV-BPIFB4 supplementation to senescent vascular cells and aged mice*. The *LAV-BPIFB4* genotype is associated with partially preserved pericyte coverage. LAV-BPIFB4 supplementation rescues human pericytes *in vitro* and improves myocardial vascular density and cardiac function in aged mice.

## 1. Introduction

Older people develop cardiac dysfunction, characterized by impaired left ventricular relaxation and contractility, coronary artery thickening and stiffness, and dysfunctional endothelium resulting in reduced coronary flow reserve.^1–5^ Structural and functional alterations are documented in cardiomyocytes, endothelial cells, and fibroblasts,^6^ and associated with microvascular rarefaction.^7^ Moreover, recent experimental evidence indicates pericyte (PC) coverage is reduced, and the cross-talk with neighbour cells is weakened in the heart and other organs of old mice.^8–11^ These defects may contribute to vascular fragility, loss of microvascular barrier integrity, and increased severity of the ischaemic injury. However, the effect of ageing on human cardiac PCs remains unknown. There is no specific treatment for halting the progression of cardiac dysfunction in elderly patients; moreover, the use of common cardiovascular medications represents a clinical challenge in this category of patients.^12^

Intrigued by the case of long-living individuals (LLIs), we have been exploring the genetic mechanisms that allow these exceptional people to avoid cardiovascular complications until the very last years of their lives.^13^ We reported that carriers of a longevity-associated variant (LAV) of the bactericidal/permeability-increasing fold-containing-family-B-member-4 gene (BPIFB4) express high levels of BPIFB4 in blood, circulating mononuclear cells, and vascular cells, and have low atherosclerotic risk.^14–16^ Moreover, *in vivo* studies demonstrated the delivery of the *LAV-BPIFB4* gene through an adeno-associated virus (AAV serotype 9) carrying a liver-specific promoter exerted broad protection in rodent models of cardiovascular disease.^17,18^ This gene transfer method allows sustained expression of secreted therapeutic proteins in the liver and systemic circulation for cross-correction of disease in other body districts.^19^ Accordingly, we showed that the cardiovascular benefit of *LAV-BPIFB4* gene delivery was mediated by molecular changes induced by the transgenic protein after its uptake by the myocardium; namely, the upregulation of contractile myosin heavy chain isoform α (MyHC-α), increased availability of stromal cell-derived factor-1 (SDF-1) and nitric oxide (NO), and activation of proteostasis.^17,18,20^

The aim of the present study was three-fold: (1) investigate the association of BPIFB4 expression, microvascular defects, and PC coverage in elderly failing human hearts; (2) determine if the exogenous provision of LAV-BPIFB4 may restore the function of cardiac PCs; and (3) finally, assess the therapeutic potential of the *LAV-BPIFB4* gene therapy in elderly mice, focusing on a potential advantage for myocardial vascularization and perfusion.

## 2. Methods

An extended Methods version is reported in Supplementary Material online. The data underlying this article will be shared upon reasonable request.

### 2.1 Immunohistochemistry study on human hearts

The study assessed the association of BPIFB4 genotypes with the PC density and coverage in aged, failing human hearts. Twenty-four patients undergoing heart transplantation for end-stage ischaemic heart failure (IHF) were enrolled at the University Hospital of Udine after signing informed consent. Controls consisted of biopsies obtained from hearts donated for cardiac transplantation (*n* = 8) or autoptic hearts collected from patients who died from causes not related to cardiovascular disease (*n* = 1). Samples were obtained from January 2016 to December 2019. The study, authorized by the local Ethics Committee (protocol no. 18386), was conducted under the declaration of Helsinki and a signed Informed Consent was collected from enrolled patients. Clinical and demographic data are reported in Supplementary material online, *Table S1*. Immunohistochemistry analyses assessed the PC density and coverage using antibodies reported in Supplementary material online, *Table S2*.

### 2.2 Molecular and cell biology studies on human cells

PCs were isolated from the explanted hearts of IHF patients (IHF-PCs, *n* = 14) and control donor hearts (C-PCs, *n* = 15) under the ethical license of the clinical study described above, according to a published protocol.^21^ Clinical and demographic data are reported in Supplementary material online, *Table S3*.

### 2.3 Gene therapy studies in mice

Experimental procedures complied with the EU Directive 2010/63/EU and principles stated in the Guide for the Care and Use of Laboratory Animals (Institute of Laboratory Animal Resources, 1996). Methods and reagents are shown in Supplementary Materials and Supplementary material online, *Table S7*.

#### 2.3.1 Gene therapy with *LAV-BPIFB4* in middle-aged and older mice

*Objective:* Two studies conducted at the University of Bristol assessed the efficacy of *AAV-LAV-BPIFB4* gene therapy in preventing cardiac dysfunction caused by ageing. *Endpoints:* Cardiac index (primary endpoint) and vascular density (secondary endpoint). *Protocol:* The protocol was approved by the British Home Office (PPL 30/3373). One week after baseline echocardiography (Vevo 3100), 14-month-old (*early intervention study:* male and female) or 18-month-old (*late intervention study:* female) C57Bl/6J mice (Charles River, Harlow, UK) were randomized to receive an AAV-vector (100 μL of a master solution containing 1 × 10^12^ GC/mL) or an equivalent volume of vehicle (PBS) (ratio of sample size = 3:1) through the tail vein, with animals under isoflurane anaesthesia (2–3%). The AAV arm comprised three subgroup treatments: *AAV9-LAV-BPIFB4*, *AAV9-WT-BPIFB4 (*the *wild-type BPIFB4* gene), or *AAV9-GFP*. Mice were examined weekly during follow-up, which was 4 months in the *early study* and 1 month in the *late study*. Subgroups underwent an additional imaging study of basal and Dobutamine-induced stress myocardial perfusion using Positron Emission Tomography (PET). Animals were terminated under isoflurane anaesthesia by exsanguination, followed by the removal of tissues and organs for histology and molecular biology.

### 2.4 Statistical analysis

The comparison of numeric variables distribution between binary variables was performed by the Student’s *t*-test or with the equivalent non-parametric test. When appropriate, one-way ANOVA (followed by Tukey’s multiple comparisons tests) or Kruskal–Wallis tests (followed by Dunn’s multiple comparison tests) were employed. Comparison among groups with two independent variables was performed utilizing two-way ANOVA followed by Sidak’s multiple comparison test. Analyses were conducted with GraphPad Prism 8.0 for MacOS or 8.4.3 for Win.

## 3. Results

### 3.1 BPIFB4 expression, capillary/arteriole density, and PC coverage in failing human hearts

**Figure 1:**
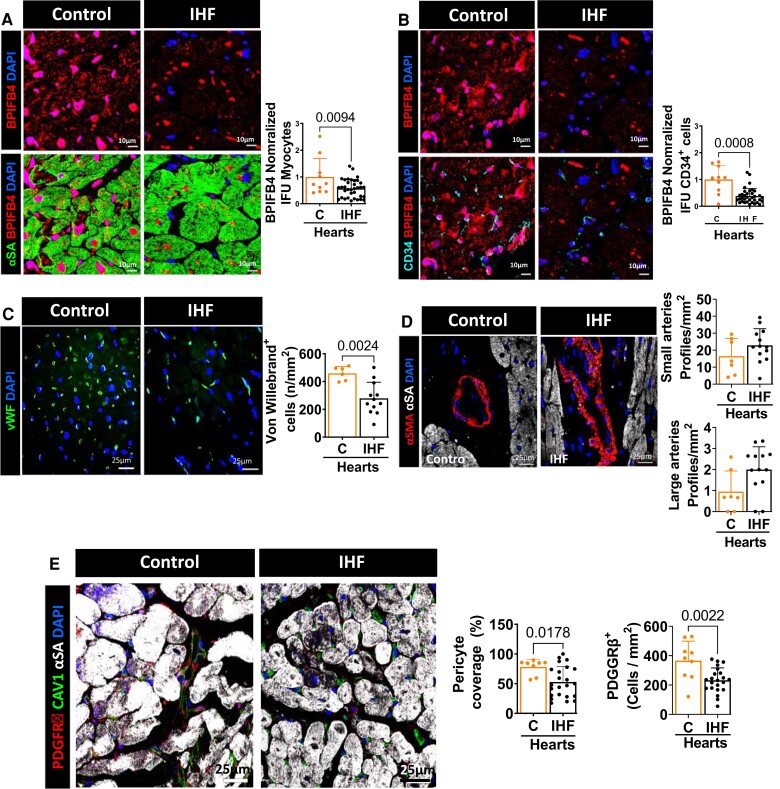
Immunohistochemical characterization of human hearts. *(A–B)* Expression of BPIFB4 in cardiomyocytes *(A)* and endothelial cells *(B)* from controls and IHF hearts. *(C–E)* Microvascular alterations in IHF hearts. Capillary density is decreased in IHF compared with control hearts *(C)*, whereas the reduction in arteriole density did not reach a statistical significance *(D)*. PC density and coverage are lower in hearts explanted from elderly patients with IHF *(E)*. PCs stained with PDGFRβ (red), endothelial with vWF or CAV1 (green) or CD34 (light blue) and cardiomyocytes with α-sarcomeric actin (αSA, green or white). Nuclei are identified by DAPI (blue) and BPIFB4 expression labelled in red. *n* = 8–9 C hearts and 23-22 IHF hearts. Data were analyzed using the Mann–Whitney *U* test (panels *B, D*, and *E*, pericyte coverage IHF vs. *C*) or unpaired Student’s *t*-test (all the other panels).

We first performed immunohistological studies on hearts explanted from elderly patients with IHF and healthy controls. As shown in Supplementary material online, *Table S1*, IHF patients were older than controls, comprised more males, and had reduced LV ejection fraction, increased heart weight, and more risk factors, including hypertension and diabetes. Interestingly, as illustrated in *Figure 1A*, IHF hearts showed lower levels of BPIFB4 in cardiomyocytes (identified by alpha sarcomeric actin, α-SA) and endothelial cells (identified by CD34). This expression defect was associated with reduced capillary density (evidenced by the number of Von Willebrand positive cells per mm^2^, *Figure 1B*), whereas arteriole density did not differ from controls (*Figure 1C*). Moreover, PC microvascular coverage and density were remarkedly reduced in IHF hearts (*Figure 1D*).

We next performed a subanalysis of data from IHF patients according to their *BPIFB4* genotype, comparing *LAV-BPIFB4* homozygous (LAV-IHF) and heterozygous or homozygous *WT-BPIFB4* (Other-IHF). As shown in Supplementary material online, *Figure S1A–C*, the expression of BPIFB4 as well as the capillary and arteriole density was similar in the two groups. However, the *LAV-BPIFB4* homozygous group had higher PC coverage and density than the other IHF hearts (see Supplementary material online, *Figure S1D*).

Together, these data indicate that older failing hearts have a deficit in BPIFB4, which is associated with the scarcity of capillaries and surrounding PCs. Moreover, although not protecting from capillary rarefaction, the LAV-BPIFB4 genotype preserved PC ensheathment of the residual microvessels.

### 3.2 Dysfunctional features of aged IHF-PCs

We next analyzed the characteristics of PCs isolated from IHF (IHF-PCs) and C hearts (C-PCs). The main clinical features of participants are reported in Supplementary material online, *Table S2*. PCs expressed the typical markers NG2, PDGFRβ, Tbx-18, and nestin. At the same time, they scored negative for PDGFRα, which characterizes cardiac myofibroblasts (see Supplementary material online, *Figure S2A*).^21^ Interestingly, aged IHF-PCs showed remarkable differences compared with C-PCs, including a 5.3-fold higher frequency of the Ki67^neg^ and γH2AX^pos^ antigenic phenotype typical of senescent cells (see Supplementary material online, *Figure S2B*),^22^ a 4.7-fold greater abundance of oxidized lipofuscin (see Supplementary material online, *Figure S2C*) and a 3.1-fold increase in mitochondrial superoxide (see Supplementary material online, *Figure S2D*), which together indicate the accumulation of biological ‘garbage’ from oxidative stress,^23^ and a 1.4-fold reduction in the nuclear location of the vitamin D receptor (VDR), which has antihypertrophic activity in the heart (see Supplementary material online, *Figure S2E*).^24^ Moreover, IHF-PCs expressed less BPIFB4 mRNA and protein (see Supplementary material online, *Figure S2F-G*).

### 3.3 In vitro LAV-BPIFB4 transfer rescues ageing PCs

**Figure 2:**
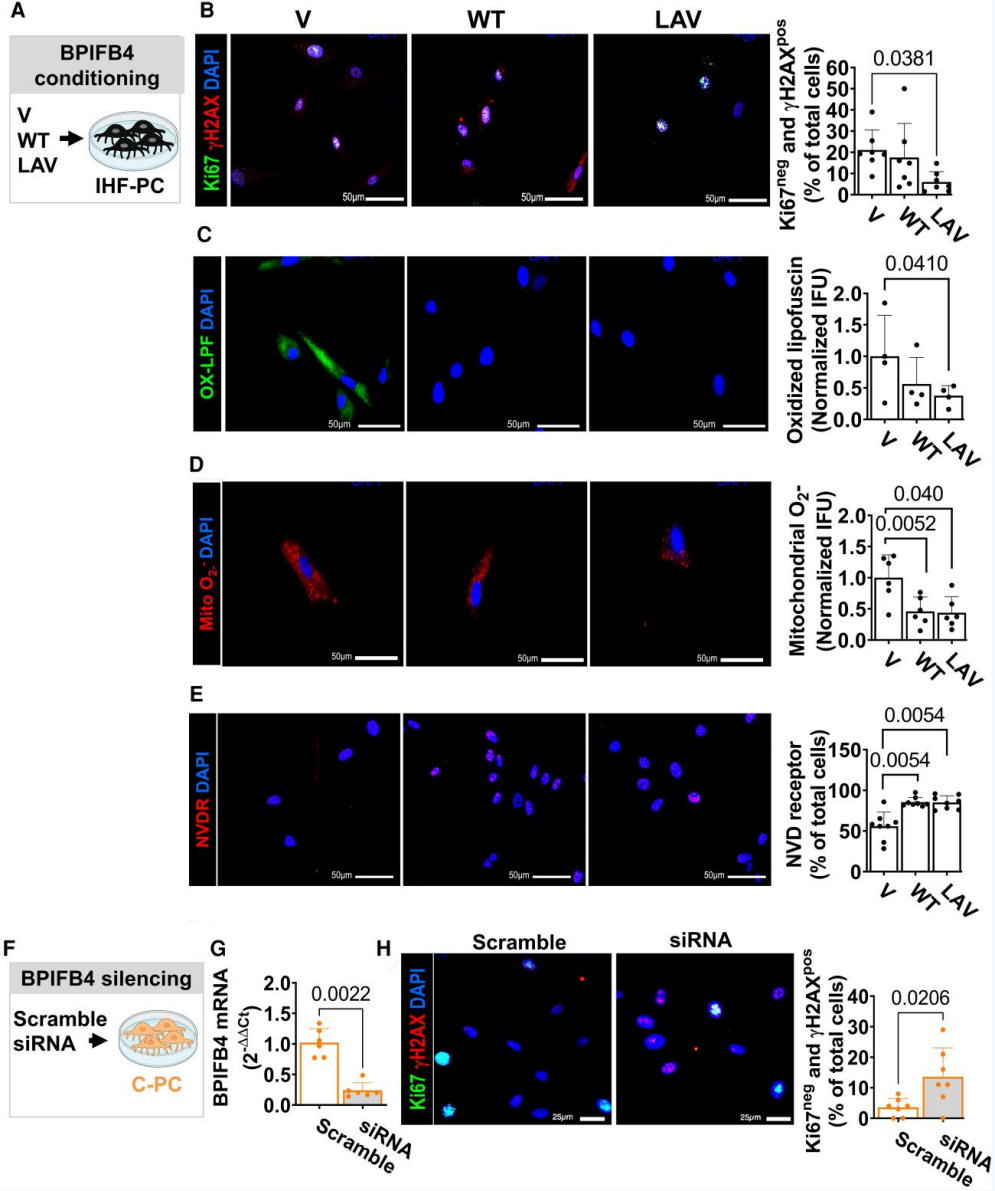
Effect of forced BPIFB4 titration on human cardiac pericytes. *(A–H)* Effects of exposing aged IHF-PCs to recombinant LAV-BPIFB4, WT-BPIFB4, or vehicle (V). The LAV-BPIFB4 protein reduced the proportion of Ki67^neg^ and γH2AX^pos^ senescent IHF-PCs (Ki67 stained green and γH2AX red) (*B*, *n* = 7 per group) and the levels of oxidized lipofuscin (green) (*C*, *n* = 4 per group). In contrast, the WT-BPIFB4 protein was ineffective in improving these endpoints. Both isoforms were similarly effective in reducing the abundance of mitochondrial O_2_^−^ radicals (*D*, *n* = 6/group) and increasing the levels of nuclear VDR [*(E)*, *n* = 8/group]. Both markers are stained red in G and H, respectively. In all the panels, nuclei are stained blue by DAPI. *(F–H)* Transfection of C-PCs with BPIFB4 siRNA or Scramble control. Effective reduction in BPIFB4 expression by siRNA (*G*, *n* = 6 per group) was associated with a significantly increased rate of Ki67^neg^ and γH2AX^pos^ senescent cells (*H*, *n* = 7 per group). Ki67 stained green and γH2AX red. Data were analyzed using ANOVA followed by Tukey's multiple comparisons test (*B, C, D* and *H*) or Kruskal–Wallis followed by Dunn's multiple comparisons test (all the other panels).

We then asked if supplementation of the recombinant LAV-BPIFB4 protein could rescue those defects (experimental protocol and treatment groups shown in *Figure 2A*). The LAV-BPIFB4 protein reduced the frequency of the Ki67^neg^ and γH2AX^pos^ PCs (*Figure 2B*) and the levels of oxidized lipofuscin compared with the vehicle (*Figure 2C*). In contrast, the WT-BPIFB4 protein was ineffective (*Figure2B* and *C*). The two BPIFB4 isoforms decreased the abundance of mitochondrial O_2_^−^ radicals and increased the fraction of VDR-expressing cells (*Figure2D* and *E*).

Next, using the opposite approach, we transfected C-PCs with a vector-based small interfering RNA (siRNA) (*Figure 2F*). As a result, BPIFB4 transcripts were remarkably reduced in C-PCs (*Figure 2G*), leading to a 3.8-fold increase in the frequency of the Ki67^neg^ and γH2AX^pos^ phenotype compared with scramble-transfected C-PCs (*Figure 2H*).

### 3.4 LAV-BPIFB4 improves the angiogenic potential of senescent ECs and IHF-PCs

**Figure 3:**
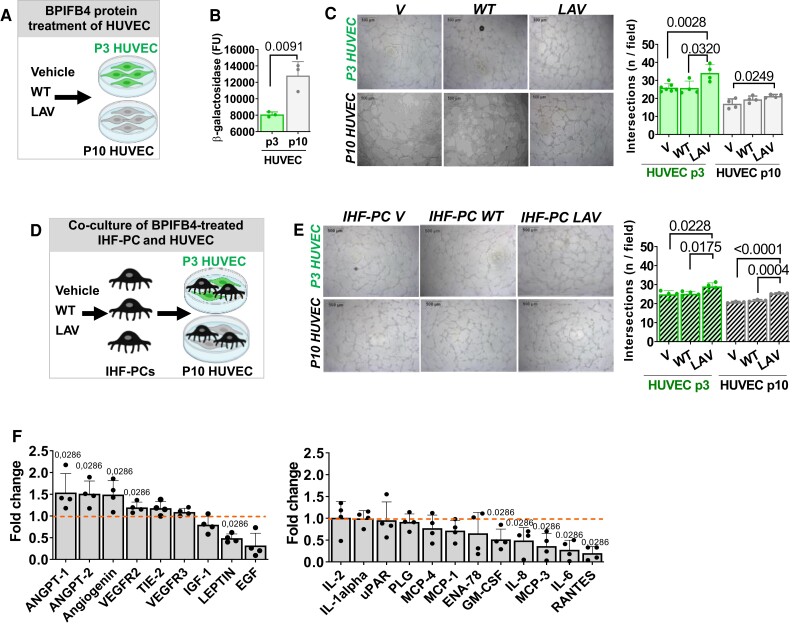
LAV-BPIFB4 enhances the ability of endothelial cells to form vascular networks in vitro. *(A)* Schematic of the BPIFB4 protein supplementation to P3 and P10 HUVEC. *(B)* Bar graph showing passaging causes proliferative senescence in HUVECs, as assessed by β-galactosidase activity (*n* = 3/group, Unpaired *t*-test). *(C)* Phase-contrast microscopy image and a bar graph showing LAV-BPIFB4 induces network formation by early and late passage HUVECs (*n* = 4/group). Data were analyzed using ANOVA followed by Tukey's multiple comparisons test). *(D)* Schematic of the experiment where the network formation assay was performed with HUVECs and aged IHF-PCs. The latter were conditioned in advance with BPIFB4 recombinant proteins. *(E)* Representative phase-contrast images and a bar graph show that aged IHF-PCs conditioned with LAV-BPIFB4 promoted networking by early and late passage HUVECs*. n* = 4 biological replicates/group. Data were analyzed using ANOVA followed by Tukey's multiple comparisons test). *(F)* Bar graph showing the levels of released angiogenic factors in the media of aged IHF-PC treated with LAV-BPIFB4 protein or vehicle. Data are expressed as fold change vs. vehicle*. n* = 4 biological replicates. Statistical analysis was performed using the Mann–Whitney *U* test.

**Figure 4:**
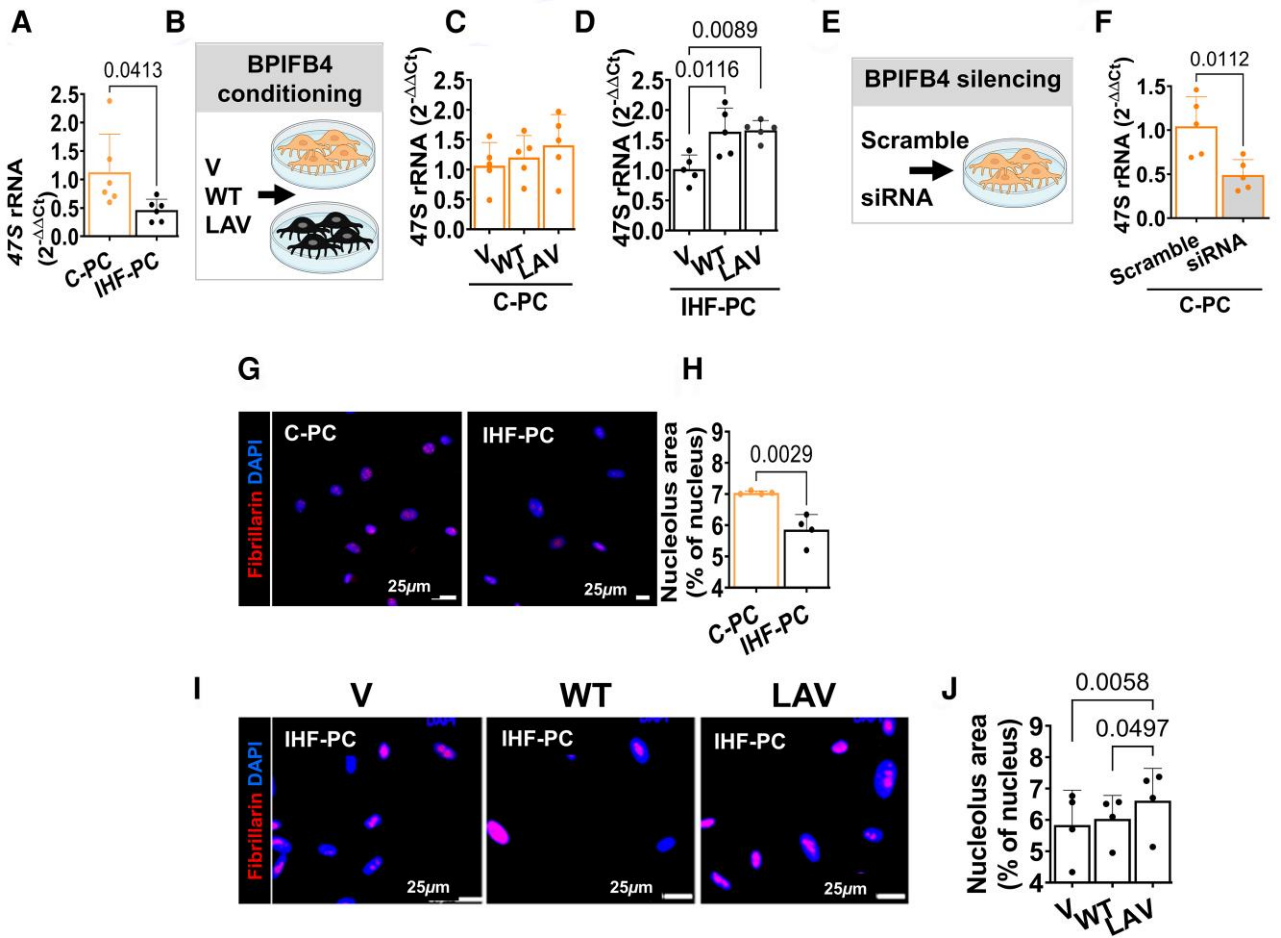
BPIFB4 promotes ribosome biogenesis. *(A–D)* Effect of BPIFB4 protein supplementation on 47S levels in PCs. *(A)* Basal levels of 47S in C-PCs and IHF-PCs (*n* = 7/group, Unpaired *t*-test). *(B)* Schematic of the conditioning experiment. *(C–D)* Supplementation of the LAV-BPIFB4 protein did not change the 47S levels in C-PCs [*(C)*, *n* = 5/group] while rescuing the 47S deficit in aged IHF-PC [*(D)*, *n* = 5/group]. ANOVA followed by Tukey's multiple comparisons test. *(E–F)* Effect of BPIFB4 silencing by siRNA on 47S in C-PC. *(F)* Depletion of BPIFB4 reduced the level of 47S in C-PC compared with scrambles compared with scramble (*n* = 5/group, Unpaired *t*-test). *(G–H)* aged IHF-PC had smaller nucleoli than C-PC (*n* = 4/group, Unpaired *t*-test). *(I–J)* This defect was rescued by LAV-BPIFB4 protein (*n* = 4/group, ANOVA followed by Tukey's multiple comparisons test). Nucleoli were stained for fibrillarin (red) and nuclei with DAPI (blue).

We next interrogated the ability of LAV-BPIFB4 to aid senescent vascular cells in forming networks in a Matrigel assay. To this aim, we first assessed the effect of LAV-BPIFB4 on early (passage 3) and late (passage 10) HUVECs (schematic in *Figure 3A*). Repeated passageing induced HUVECs to become senescent, as indicated by a 1.6-fold increase in β-galactosidase activity (*Figure 3B*). LAV-BPIFB4 protein supplementation enhanced the ability of early and late passage HUVECs to form networks on Matrigel compared with corresponding HUVEC controls stimulated with WT-BPIFB4 or vehicle (*Figure 3C*). Similarly, LAV-BPIFB4 conditioning of aged IHF-PCs increased their capacity to support the formation of networks made of early or late passage HUVECs (*Figure3D* and *E*) and caused significant changes in aged IHF-PC secreted proteins, increasing pro-angiogenic factors and decreasing pro-inflammatory factors (*Figure 3F* and see Supplementary material online, *Figure S3A*). In additional experiments, we verified that, while not affecting cell viability, LAV-BPIFB4 supplementation restored the impaired migration capacity of late passage HUVECs (see Supplementary material online, *Figure 4A* and *B*).

These *in vitro* data suggest that LAV-BPIFB4 can improve the depressed angiogenic activity of senescent endothelial cells both directly and through paracrine inputs from PCs.

### 3.5 LAV-BPIFB4 induces rRNA transcription and ribosomal biogenesis

Perturbations of the circuitry between nucleolar activity, rRNA transcription, and translation lead to ageing-related cell deterioration. In line with this, BPIFB4-deficient older IHF-PCs showed lower levels of precursor 47S rRNA transcripts than C-PCs, suggesting depressed transcription or heightened degradation of the primary transcript (*Figure 4A*). The supplementation with recombinant LAV-BPIFB4 or WT-BPIFB4 proteins selectively increased the 47S RNA levels in aged IHF-PCs but not in C-PCs (*Figure 4B**–**D*). Conversely, BPIFB4 abrogation in C-PCs decreased the transcription of 47S compared with scramble controls (*Figure4E* and *F*).

The nucleolus of aged IHF-PCs was smaller than that of C-PCs (*Figure4G* and *H*), which may be compatible with cellular stress impacting the transcriptional machinery. This feature was corrected by LAV-BPIFB4 supplementation (*Figure4I* and *J*).

### 3.6 LAV-BPIFB4 interacts with nucleolin to support angiogenesis

Nucleolar proteins, such as nucleolin (NCL), known to modulate ribosome biogenesis and DNA repair,^25^ are also essential for endothelial cell migration and tubule formation.^26^ Data from the Nuclear Receptor Signaling Atlas consortium (NURSA; http://www.nursa.org) showed that BPIFB4 and NCL are enriched within an interactive multi-protein complex.^27^ This finding made us consider whether NCL could partner with BPIFB4 in promoting angiogenesis.

**Figure 5:**
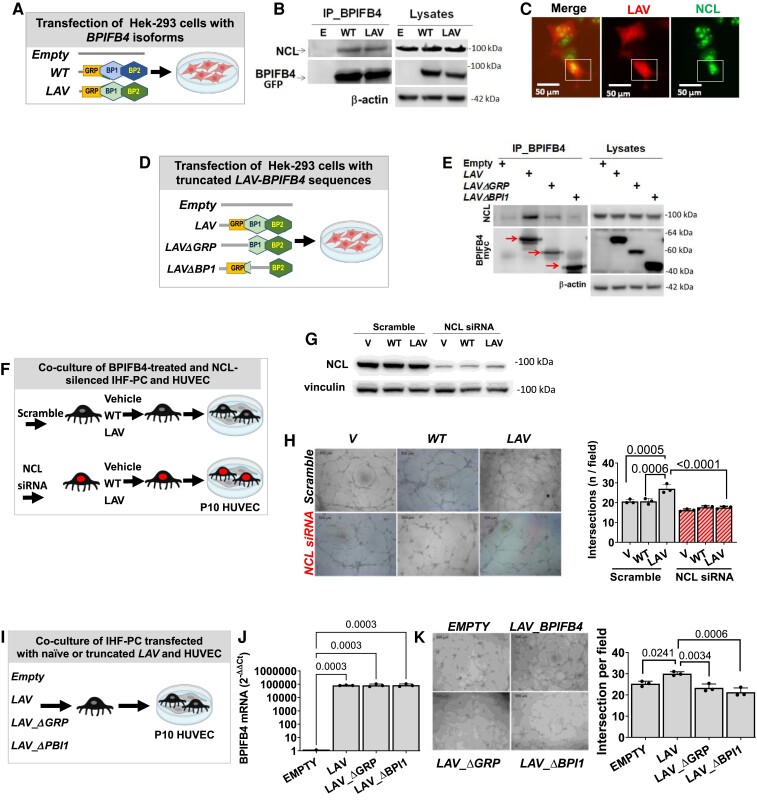
LAV-BPIFB4 physically interacts with NCL improving angiogenesis. The binding between BPIFB4 and NCL was determined using co-immunoprecipitation assays. *(A)* Cartoon showing the BPIFB4 transfection groups. *(B)* Lysates from Hek-293 transfectants expressing the BPIFB4 isoforms were immunoprecipitated with anti-BPIFB4, resolved by SDS-PAGE (10%), and probed with anti-NCL antibody (left panel). Lysate aliquots were loaded to verify transfection and immunoprecipitation efficiencies (right panel). *(C)* The subcellular localization of exogenous LAV-BPIFB4 and endogenous NCL in transfected Hek-293 was determined using double staining immunofluorescence using anti-BPIFB4 polyclonal (red) and anti-NCL monoclonal (green) antibodies. White squares point to the punctate area of colocalization between LAV-BPIFB4 and NCL. *(D)* Schematic representation of BPIFB4 constructs used for transfection. *(E)* Lysates from Hek-293 transfectants expressing deleted forms of LAV-BPIFB4 were immunoprecipitated with anti-BPIFB4 antibody, resolved by SDS-PAGE (10%), and probed with indicated antibodies (upper panel). *(F)* Schematic of the experiment in which IHF-PCs were silenced with siRNA against NCL or scramble siRNA and then exposed to the conditioning with BPIFB4 recombinant proteins before undergoing the Matrigel assay with late passage HUVECs. *(G)* Confirmation of effective silencing of NCL by siRNA. Western blotting and bar graph showing the data of different groups (*n* = 3 biological replicates/group). *(H)* Representative phase-contrast images of the six experimental groups. Bar graph showing that NCL silencing abolished the pro-angiogenic effect of LAV-BPIFB4 conditioned aged IHF-PCs (*n* = 3 biological replicates/group, ANOVA followed by Tukey's multiple comparisons test). *(I)* Schematic of the experiment in which IHF-PCs were transfected with the whole LAV-BPIFB4 sequence or truncated mutants impeding the interaction of the encoded protein with NCL. *(J)* Effective expression of the transgenes is shown at the mRNA. ANOVA followed by Tukey's multiple comparisons test. *(K)* Representative phase-contrast images of the six experimental groups. Bar graph showing that IHF-PCs transfected with the whole LAV-BPIFB4 sequence have a pro-angiogenic capacity, which is negated to aged IHF-PC transfected with the truncated mutants (*n* = 3 biological replicates/group, ANOVA followed by Tukey's multiple comparisons test.

To validate this interaction, a co-immunoprecipitation assay was performed in Hek-293 cells transfected with BPIFB4 isoforms (WT or LAV) or empty (*Figure 5A*). After lysate immunoprecipitation using a polyclonal anti-BPIFB4 antibody, we assessed the protein interaction using western blots. NCL coimmunoprecipitates with the tested BPIFB4 isoforms (*Figure 5B*). This interaction was confirmed on the same coimmunoprecipitates using mass spectrometry (data not shown) and further validated through fluorescence microscopy colocalization of the two proteins in the nucleus/nucleolus of transfected Hek-293 cells (*Figure 5C*).

To further identify the BPIFB4 structure element required for the interaction with NCL, we transfected Hek-293 cells with a series of truncated forms of *BPIFB4* lacking putative binding sites for NCL (*Figure 5D*). Deletion of up to the amino acid (AA) 103 corresponding to the glycine-rich peptide (GRP) (*LAV_ΔGRP construct*) resulted in weaker binding to NCL (*Figure 5E*). Further truncation up to AA 197, relative to the first Bactericidal permeability-increasing protein (BPI) (*LAV_ΔBPI1 construct*), strongly compromised the protein–protein interaction (*Figure 5E*).

Interestingly, NCL silencing using a siRNA vector (*Figure5F* and *G*) inhibited the pro-angiogenic action of LAV-BPIFB4-treated aged IHF-PCs (*Figure 5H*). The role of NCL was further strengthened by an experiment where aged IHF-PCs were transfected with LAV-BPIFB4 constructs with or without the binding sequence for NCL and then cocultured with HUVECs in the Matrigel assay (*Figure5I* and *J*). Interestingly, LAV-BPIFB4-transfected IHF-PCs encouraged late passage HUVECs to form networks, whereas those transfected with the LAV-BPIFB4 construct lacking the NCL binding sequence were ineffective (*Figure 5K*). These data indicate that LAV-BPIFB4 synergically works with NCL to regulate PC-induced angiogenesis.

### 3.7 *LAV-BPIFB4* gene therapy protects the heart from ageing

**Figure 6:**
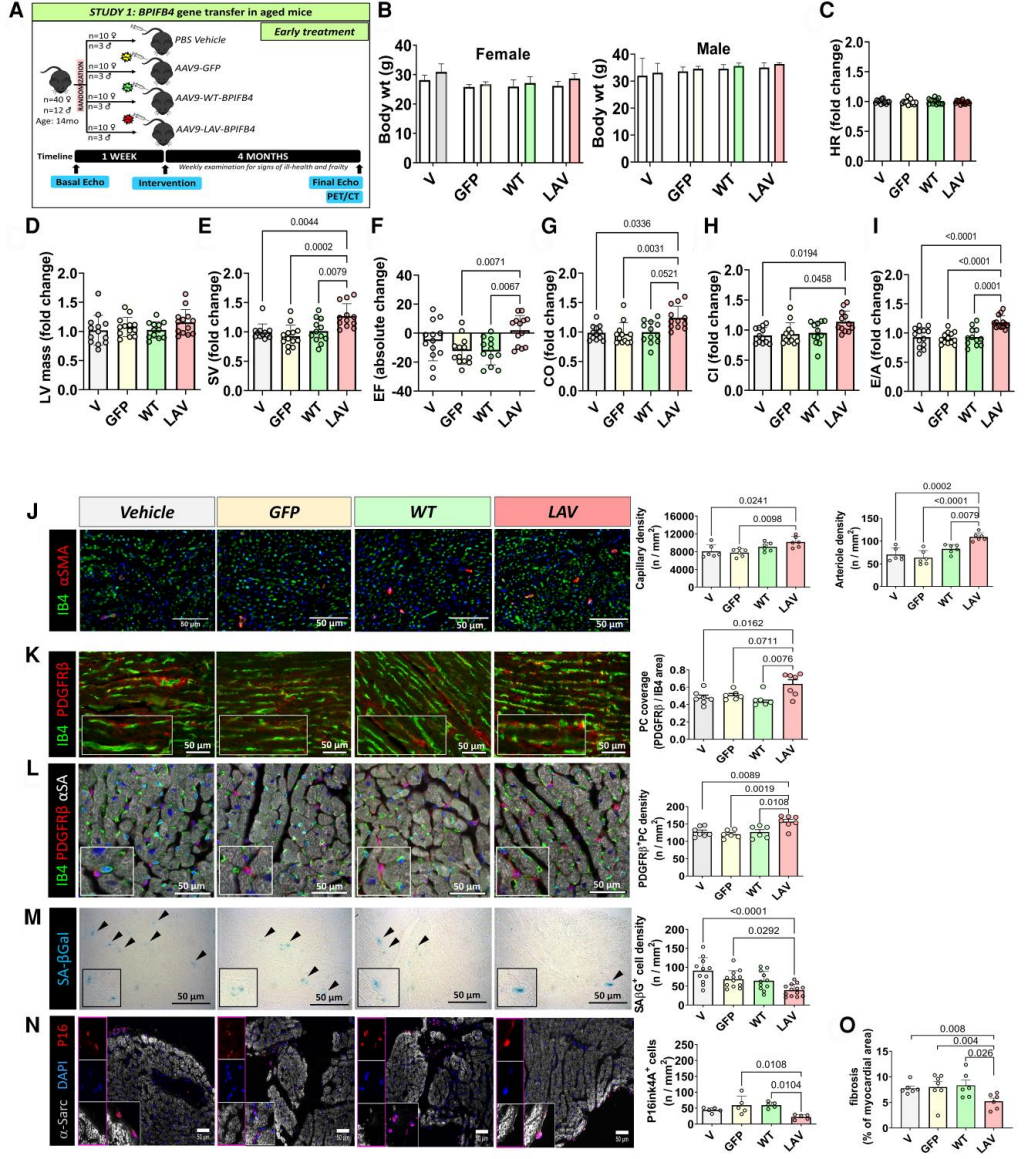
Early LAV-BPIFB4 gene therapy improves cardiac function in elderly mice. *(A)* Cardiac function was assessed in female mice at baseline (14 months old) and 4 months post-treatment (18 months old). *n* = 10 female and 3 male mice/group. *(B)* Body weight at baseline and the end of follow-up. *(C–I)* Functional parameters are all expressed as fold change from baseline except for Ejection Fraction which is illustrated as absolute change from baseline. Heart rate (HR) *(C)*, left ventricular mass *(D)*, stroke volume (SV) *(E)*, ejection fraction (EF) *(F)*, cardiac output (CO) (*G)*, cardiac index (CI) *(H)*, and *E/A* ratio *(I)*. Bar graphs show combined data for male and female mice, including the mean, standard deviation, and individual values. *(J–L)* Histological analysis of the vascular density in hearts harvested at the end of the follow-up. Representative images of isolectin B4 (green) positive endothelial cells, α-smooth muscle actin (red) positive smooth muscle cells *(J)*, and PDGFRβ (red) positive PCs *(K–L)* with nuclei identified by DAPI (blue) in heart sections from mice attributed to the 4 groups. Scale bars: 50 μm. Graphs showing the density of capillaries and arteries *(J)*, and pericyte coverage *(K)* and density *(L)*. *n* = 6 mice/group. *(M–N)* Representative images and bar graphs showing the density of senescence-associated β-galactosidase *(M)* or p16ink4A *(N)* positive cells in sections from hearts of mice attributed to the 4 groups. Scale bars: 50 μm. *n* = 5 to 13 mice/group. *(O)* Cardiac fibrosis was assessed by staining with Azan Mallory in female mice at 4 months post-treatment (18 months old). *n* = 6–7 mice/group. Data were analyzed using parametric ANOVA followed by Tukey's multiple comparisons test, except for panel *E*, *G*, *H*, and *I* where the Kruskal–Wallis test was applied followed by Dunn's multiple comparisons test.

In a study on middle-aged mice of both sexes (*early study* illustrated in *Figure 6A*), neither *LAV-BPIFB4* nor control treatments affected animals’ body weight (*Figure 6B*). At baseline, all groups showed similar echocardiography parameters. No group difference was observed regarding HR and LV mass before and after treatment (*Figure6C* and *D* and Supplementary material online, *Figure S5B* and *C*). Notably, the *LAV*-treated group showed better indexes of LV function, including a higher stroke volume (as compared with its baseline or other treatments’ effect), preserved ejection fraction, which was instead reduced from baseline to final measurement in the other groups, and improved cardiac output and cardiac index as compared with corresponding baseline values or other treatments (*Figure 6E**–**H* and Supplementary material online, *Figure S5D–I*). Basal *E/A* data denoted a mildly compromised diastolic function in middle-aged mice, being on average less than 1.4. This deficit was improved by the *LAV-BPIFB4* treatment (*Figure 6I* and Supplementary material online, *Figure S5J*). The reported benefits were confirmed after considering the influence of sex as a confounder.

Immunohistochemistry demonstrated the increased staining for BPIFB4 in cardiac tissue from the *LAV*-treated group where the protein localized in myocytes and vascular cells (see Supplementary material online, *Figure S6A* and *B*). Moreover, capillary and arteriole density were increased in cardiac sections of mice treated with *LAV* compared with controls (*Figure 6J*), this effect is associated with higher PC coverage and density (*Figure6K* and *L*). Senescent cells, identified by the expression of β-galactosidase or p16Ink4A, were mainly located in the interstitial space, and their frequency was reduced in the group treated with *LAV* (*Figure6M* and *N*). This was in keeping with the reductive effect of *LAV* on the expression of the histone H3.3 and γH2AX in the mouse heart (see Supplementary material online, *Figure S7*). Moreover, *LAV* treatment reduced myocardial fibrosis (*Figure 6O*).

**Figure 7:**
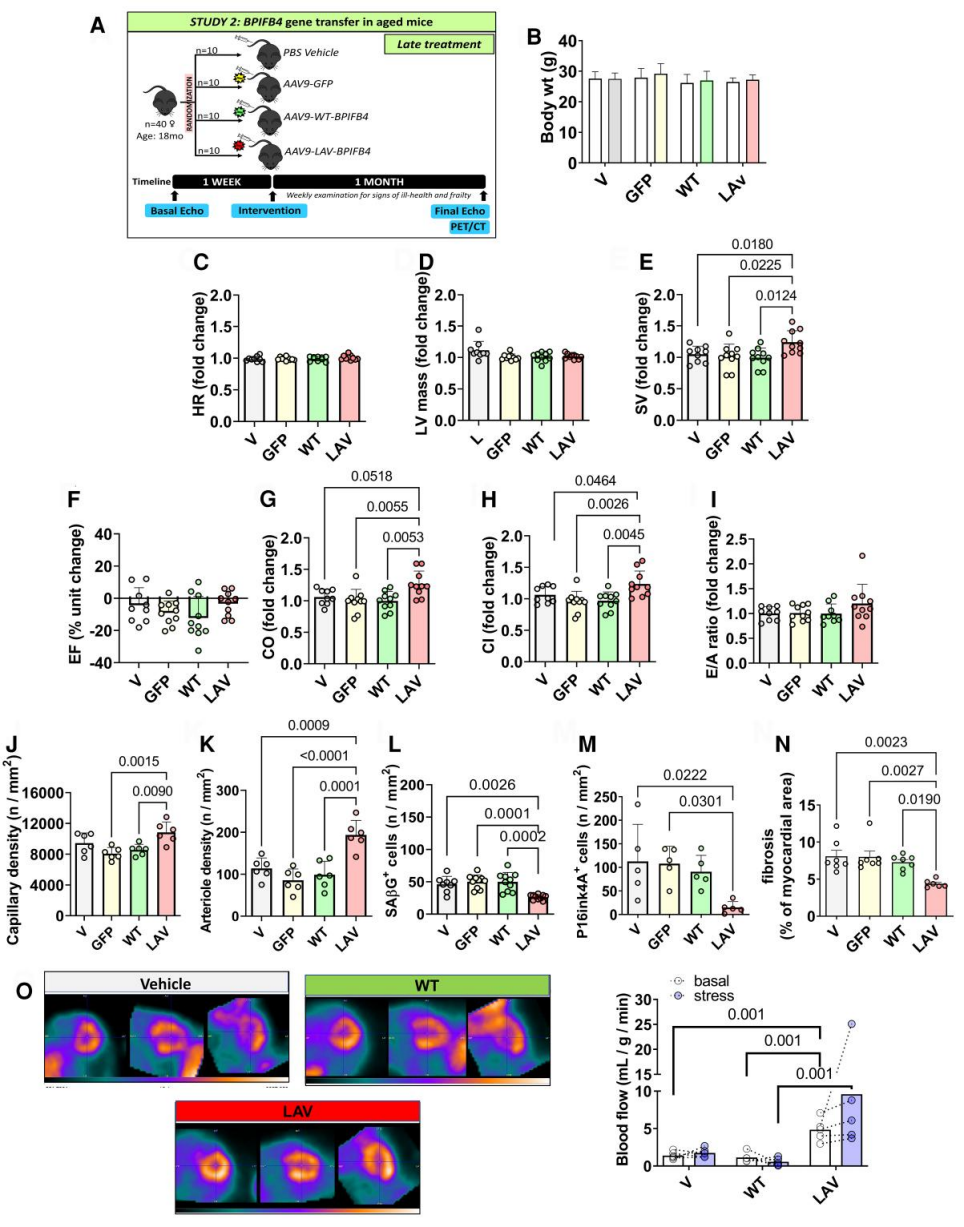
Late LAV-BPIFB4 gene therapy improves cardiac function in elderly mice. *(A)* Cardiac function was assessed in female mice at baseline (18 months old) and four weeks post-treatment (19 months old). *n* = 10 female mice per group. *(B)* Body weight. *(C–I)* Fold changes in functional parameters from basal measurements except for Ejection Fraction, which is expressed as absolute unit change. Heart rate (HR) *(C)*, left ventricular mass *(D)*, stroke volume (SV) *(E)*, ejection fraction (EF) *(F*), cardiac output (CO) *(G)*, cardiac index (CI) *(H)*, and *E/A* ratio *(I)*. *(J–K)* Graphs show capillaries *(J)* and arteries *(K)* density. *n* = 6 mice per group. *(L–M)* Graphs showing data of the density of β-galactosidase *(L)* and p16ink4A *(M)* positive cells. *n* = 5 to 10 mice per group. *(N)* Cardiac fibrosis was assessed after staining with Azan Mallory in female mice at 1-month post-treatment (19 months old). *n* = 6–7 mice per group. *(O)* Representative images of PET imaging were performed in subgroups of the early and late intervention studies. Representative images. The bar graph shows the data from basal and Dobutamine stress tests. *n* = 5 mice per group. Values are presented as mean, standard deviation, and individual values. Data were analyzed using ANOVA followed by Tukey's multiple comparisons test, except for panels *D* and *G* where the Kruskal–Wallis test was applied followed by Dunn's multiple comparisons test.

A second study was conducted on elderly mice (*late study* illustrated in *Figure 7A*). Mice had lower baseline cardiac index values than mice of the *early study* (0.46 vs. 0.51, *P* < 0.05) and a trend toward a further reduction in *E/A* (1.33 vs. 1.38, *P* = 0.15), confirming that the former had more advanced cardiomyopathy as expected, given their older age.

There was no group difference in weight gain, HR, and LV mass throughout the study (*Figure 7B**-**D*). As in the *early study*, *LAV* but not *WT, GFP*, or vehicle maintained or improved the parameters of systolic function (*Figure 7E**–**H* and see Supplementary material online, *Figure S8*). Regarding the *E/A* ratio, the *LAV* group showed higher final values than the controls (see Supplementary material online, *Figure S8J*). Yet, the fold change of the *E/A* ratio from basal to the last measurement was similar between groups (*Figure 7I*). Histological examination of the hearts showed that animals given *LAV* had increased vascular density at both capillary and arteriole levels (*Figure7J* and *K*) while showing decreased numbers of senescent cells in the cardiac interstitial space (*Figure7L* and *M***)**. LAV treatment reduced myocardial fibrosis (*Figure 7N*). Apoptotic events were rare in the vehicle group (<1% of total cells), and no change was observed in any treatment (data not shown).

### 3.8 LAV-BPIFB4 gene therapy improves myocardial perfusion

Finally, mice from the *early* and *late* studies were assessed using PET/CT imageing. As shown in *Figure 7O*, *LAV* increased basal and Dobutamine stress-induced myocardial perfusion. Dobutamine is a β_1_-adrenergic agonist widely used as a pharmacological stressor to assess left ventricular wall motion and myocardial perfusion.^28^ It was also used in mice to characterize systolic and diastolic function in normal and chronically failing hearts during inotropic stimulation.^29^ The enhancement of the diastolic component of the flow determined by dobutamine, dipyridamole, or adenosine can be used to calculate the coronary flow velocity reserve.^30,31^ Therefore, our data suggest that the LAV treatment may have restored coronary blood flow response to adrenergic stimulation, which is decreased in older people and patients with heart failure due to the downregulation of the β_1_-adrenergic receptor subtype.^32^

## 4. Discussion

The present study integrates multiple, mutually supporting lines of evidence for the protective role of LAV-BPIFB4 against age-related heart disease: (i) an association between BPIFB4 expression, microvascular density, and pericyte ensheathment in the human heart; (ii) a remarkable benefit of LAV-BPIFB4 supplementation on senescent vascular cells; and (iii) a preventive and therapeutic action of *LAV-BPIFB4* gene therapy in animal models of cardiac ageing.

### 4.1 Reduced BPIFB4 expression in the failing human heart

Using immunohistochemistry studies, we demonstrated that elderly failing human hearts have a reduced expression of BPIFB4 in cardiomyocytes and endothelial cells, accompanied by microvascular rarefaction and reduced PC density and coverage. This novel finding strengthens the data from a recent experimental study in elderly mice showing cardiac PC loss and downregulation of AKT phosphorylation, a well pro-survival and pro-angiogenic kinase downstream to the PDGFRβ receptor.^11^ Notably, IHF patients homozygous for the *LAV-BPIFB4* genotype were seemingly spared from the PC defect, possibly representing an extreme homeostatic response overwhelmed by the end-stage disease.

### 4.2 LAV-BPIFB4 rescues the pro-angiogenic activity of cardiac pericytes from elderly failing human hearts

LAV-BPIFB4 supplementation reduced the expression of senescence markers and improved angiogenic functions of PCs from aged, failing human hearts. Interestingly, LAV-BPIFB4 caused a remarkable shift in the paracrine repertoire of aged IHF-PCs, heightening the secretion of angiogenic factors and reducing the release of inflammatory cytokines. This paracrine response, together with the direct action of the LAV-BPIFB4 protein on endothelial cells, accounts for the improvement of network formation observed in coculture experiments *in vitro*. Another novel finding from studies on senescent PCs consists of the LAV-BPIFB4 favourable effect on rRNA transcription and ribosomal biogenesis, which, together with RNA-binding protein activity and protein translation, play fundamental roles during angiogenesis.^33^

### 4.3 NCL provides a subcellular platform for LAV-BPIFB4 to induce transcriptional regulation of angiogenesis

Dysregulation in the manufacture of ribosomes accelerates cellular ageing. Data in aged IHF-PCs indicate that both BPIFB4 isoforms promote rRNA transcription, although the outcome was more prominent with LAV-BPIFB4. This information complements our previous report regarding the capacity of ectopic BPIFB4 to induce several small nucleolar RNAs involved in the modification, maturation, and stabilization of rRNA and pre-rRNA cleavage.^17^

We newly show a functional partnership between LAV-BPIFB4 and NCL, which was hinted at by a previous co-immunoprecipitation study from the NURSA consortium.^27^ NCL plays critical roles in lifespan extension, and DNA repair,^34,35^ regulates ageing and cellular plasticity in cardiomyocytes,^36,37^ and protects against ischaemia-induced damage through macrophage polarization and potentiation of angiogenic and anti-apoptotic pathways.^38–40^ Expanding this knowledge, we showed that silencing NCL or deleting the LAV-BPIFB4’s binding sequence to NCL impeded LAV-BPIFB4 to stimulate aged IHF-PCs in gaining the capacity to promote endothelial networks. This data suggests NCL mediates the pro-angiogenic action of LAV-BPIFB4 through transcriptional activation of angiocrine mediators. Interestingly, recent evidence from co-immunoprecipitation studies indicated that NCL potentiates the SDF-1/CXCR4 axis by activating signalling downstream to the receptor.^41,42^ We also reported that activation of the SDF-1/CXCR4 signalling pathway plays a role in the cardiovascular protective effects of LAV-BPIFB4 gene therapy in type 2 diabetic and atherosclerotic mice.^16,18^

### 4.4 LAV-BPIFB4 gene therapy protects the ageing heart

We have previously shown that the systemic delivery of *LAV-BPIFB4 via* the AAV-9 vector results in the expression of the encoded protein in the heart of diabetic mice.^18^ Here, we provide additional evidence for the upregulation of the LAV-BPIFB4 protein by cardiac vascular cells. Elderly people have a reduced exercise tolerance and a decreased left ventricle inotropic and perfusion reserve. In addition, adrenergic responsiveness is altered with ageing.^43^ In the early study, we showed that *LAV-BPIFB4* gene therapy benefited systolic and diastolic function as well as basal perfusion and coronary flow response to β1 adrenergic stimulation (Dobutamine test). The late study, where *LAV-BPIFB4* was delivered to 18-month-old mice, confirmed the improvement of systolic function and coronary flow reserve, which was restored to levels like those recorded in middle-aged mice. In contrast, the benefit of the diastolic index *E/A* was less evident. Translated to the human condition, the recovery of contractility indexes seen in older mice would correspond to rewinding the heart’s biological clock by more than ten years. Moreover, the response of coronary blood flow to Dobutamine suggests that LAV treatment can restore cardiac β-adrenergic responsiveness in the ageing heart.

### 4.5 Anatomical basis of LAV-BPIFB4-induced protection on the ageing heart

The decline of cardiac function with ageing is due to the combination of cardiomyocyte senescence and death, vascular rarefaction, and interstitial fibrosis. *LAV-BPIFB4* therapy impacted these alterations, improving vascularization and PC coverage, and reducing cell senescence and collagen accumulation in the ageing murine heart. Moreover, we have previously shown that PCs have regenerative potential, related partly to their paracrine action on endothelial cells and cardiomyocytes, and the capacity to differentiate into a contractile phenotype inducive of arteriogenesis.^44,45^ We speculate that these mechanisms may be restored in rejuvenated cardiac PCs.

### 4.6 Study limitations and conclusions

In this study, we exploited the genetics of longevity to revitalize the function and vascularization of ageing hearts in mice and the angiogenic properties of cardiac PCs from aged failing human hearts. More investigation is needed to determine the duration of the *in vivo* therapeutic effect and the necessity of repeated administrations. It remains to be ascertained whether the benefit observed in mice can be translated into therapeutic results at advanced stages of heart failure. Additional efficacy/safety studies toward regulatory approval of the longevity gene/protein will determine if this new technology can introduce a change in the prevention and treatment of age-related disease, restoring health rather than amending the damage inflicted by ageing.

## Supplementary Material

cvad008_Supplementary_Data

## Data Availability

All data associated with this study are in the paper. A detailed description of the methods is available in the manuscript, Supplementary information on source data. Source data are provided in this paper.

## Notes

Translational perspective New treatments capable of delaying the heart’s spontaneous ageing are urgently needed. Genetic determinants of healthy longevity are attractive druggable targets for two reasons. First, they impinge upon the biological clock at a multi-organ level. Second, they have already been validated through natural selection in humans, which increases confidence about the clinical efficacy and safety of the derived medicinal products. These findings, based on the use of gene transfer and recombinant protein, pave the way for the employment of LAV-BPIFB4 to reverse the decline of heart performance in older people.
