# Supplementary material for: The longevity-associated BPIFB4 gene supports cardiac function and vascularization in ageing cardiomyopathy
Source: Cardiovasc Res. 2023 Jan 13;119(7):1583–95. doi: 10.1093/cvr/cvad008 (PMC10318395; doi:10.1093/cvr/cvad008)
Supplement: cvad008_Supplementary_Data [file cvad008_supplementary_data.docx]

**EXPANDED ONLINE METHODS**

**PC density and coverage in the heart of elderly patients with IHF**

Twenty-four patients undergoing heart transplantation for end-stage IHF were enrolled at the University Hospital of Udine after signing informed consent. Controls consisted of biopsies obtained from hearts donated for cardiac transplantation (n=8) or autoptic hearts collected from patients who died from causes not related to cardiovascular disease (n=1). **Supplementary Table 1** illustrates the main clinical and demographic data. Once harvested from organ donors, hearts were either transported employing St. Thomas’ cardioplegia infusion and cold storage (CS) in ice, or ex-vivo perfused (EVP) employing the organ care system (OCS) device (TransMedics Inc., Boston, MA). At the end of the transplantation procedure, a fragment of the atrium of the donated heart and the whole explanted failing heart were sent to the pathology department for histology and cell studies. Donor heart tissue and the failing heart were kept at +4°C until further processing. Tissue sampling and cell isolation for primary cultures of cardiac PCs (for both donor and explanted patients) were carried out within about 3 h after receiving the samples. Macroscopic examination, tissue sampling, and histology were conducted by an expert pathologist (Nicoletta Finato). **Supplementary Table 2** summarizes the conditions employed for immunofluorescence assays

**Cell isolation and culture**

PCs were isolated from the IHF hearts (IHF-PCs, n=14) and control (C-PCs, n=15) following an isolation/expansion protocol reported previously.^1^ Clinical data of the subjects are summarized in **Supplementary Table 3**. The purity of the cell preparations was confirmed by analysis of typical PC markers using immunocytochemistry, as previously described.^2^ All the *in vitro* assays were conducted in PCs between the 3rd and 4^th^ passage.

Human umbilical vein endothelial cells (HUVECs) were purchased from Invitrogen (Inchinnan, Scotland) and cultured in Vascular Cell Based Medium (ATCC, Tell City, USA), supplemented with endothelial cell growth kit-VEGF (ATCC). All the *in vitro* assays were conducted in HUVECs at the 3rd and 10^th^ passage. Hek-293 (ATCC) were cultured in DMEM supplemented with 10% foetal bovine serum (FBS) (Thermo Fisher Scientific, Waltham, USA)

**Production of *Bpifb4*** **vectors and recombinant Bpifb4** **proteins**

LAV-∆GRP- and LAV-∆BPI1-BPIFB4 were identified by RT-PCR using cDNA isolated from iPSCs (Thermo Fisher Scientific). Primers were designed in the 5’- and 3’- UTR of BPIFB4 and listed in **Supplementary Table 4** (named BPIFB4^A^)**.** The amplified PCR products were cloned in pGEM-T Easy vector system (Promega, Madison, USA) and verified by sequencing. The WT- BPIFB4, LAV-BPIFB4-pRK5 constructs were previously described.^3^ LAV-BPIFB4, LAV-∆GRP, and LAV-∆BPI1-BPIFB4 cDNAs were subcloned into SgfI-MluI digested pCMV6-Entry vector (Origene, Rockville, USA) using primers listed in Supplementary Table 4 (named BPIFB4^B^).

The adeno-associated vector (AAV) was produced as described previously.^3^ For each viral preparation, physical titers (GC/mL) were determined through dot-blot analysis and polymerase chain reaction quantification using TaqMan20 (Applied Biosystems, Carlsbad, USA).

Recombinant BPIFB4-His protein expression and purification were previously described.^4^ Protein samples purified from Hek-293 transfected with the empty vector was used as a negative control and referred to as vehicle. The protein concentration was determined using a Qubit fluorometer (Thermo Fisher Scientific).

**Quality control of purified recombinant proteins**

Aliquots of the protein samples purified from Hek-293 transfected with empty (vehicle) WT- and LAV-BPIFB4 vectors were separated by electrophoresis using 4–12% NuPAGE Bis-Tris protein gels and probed with antibody anti-BPIFB4 (**Supplementary Figure 9**).

**Protein conditioning**

Cells were conditioned with WT- and LAV-BPIFB4 recombinant proteins, or vehicle (20 ng/ml) for 24 h before performing functional assays.

**Transfections**

Cells were transfected with the indicated plasmids using Lipofectamine 2000 (Invitrogen) according to the manufacturer’s instructions and harvested 48 h after transfection.

**RNA silencing**

Specific ON-TARGET plus SMART pool siRNA (Dharmacon, Horizon, Cambridge, UK) were used to knock down *Bpifb4* and *NCL* mRNA expression. Cells were transfected at 70% of confluence with 30 nM siRNA-BPIFB4 or with 30 nM Negative Control (ON-TARGET plus Non-Targeting pool - Dharmacon) using Lipofectamine RNAiMAX (Thermo Fisher Scientific), according to the manufacturer’s instructions, and harvested 72 h after transfection. PCs were transfected twice with 10 nM siRNA-NCL or with 10 nM negative control using Lipofectamine RNAiMAX, according to the manufacturer’s instructions, and harvested 48 h Cafter transfection.

**RNA Extraction and Quantitative Real-Time Analysis**

RNA was extracted with RNeasy (Qiagen, Germantown, USA), following the protocol provided by the manufacturer. Total RNA concentration and quality were determined using a Nanodrop spectrophotometer (Nanodrop 1000, Thermo Fisher Scientific). Before retro-transcription, DNAse I (Thermo Fisher Scientific) was used to remove genomic DNA contamination. Subsequently, Superscript III, Oligo(dT)12-18, dNTPs mix, and RNaseOUT (Thermo Fisher Scientific) were used to synthesize cDNA, following the manufacturer’s protocol. QuantStudio^TM^ 6 Flex Real-Time PCR System (Applied Biosystems) and SYBR Green PCR Master Mix (Applied Biosystems, Life Technology) were employed to conduct Real-Time-qPCR analyses, on triplicate samples of retrotranscribed cDNA. Expression levels were normalized to GAPDH. Primer sequences are listed in **Supplementary Table 4.** Data were expressed as 2-(ΔΔCt)

**Western blotting**

Cells were lysed in RIPA buffer containing protease and phosphatase inhibitor cocktail (Sigma-Aldrich). Protein concentration was determined using the Bradford assay (Sigma-Aldrich). Total proteins were separated by electrophoresis using 4–12% NuPAGE Bis-Tris protein gels (Thermo Fisher Scientific), transferred onto a polyvinylidene difluoride (PVDF) membrane (GE Healthcare, Buckinghamshire, UK), and probed using the conditions indicated in Supplementary Table 5. Blots were revealed by Western Sure Premium Chemiluminescent Substrate LI-COR and by a C-DiGit Blot scanner (LI-COR Biosciences, Lincoln, USA). Densitometric quantifications were normalized relative to beta-Actin signal using Image Studio software (<http://www.licor.com)>.

**Immunoprecipitation**

Lysates obtained from Hek-293 cells transfected with indicated plasmids were precleared and incubated with anti-BPIFB4 antibody (Clinsciences, custom made, Guidonia Montecello, Italy) immobilized on protein A-Sepharose (Thermo Fisher Scientific). The precipitates were washed twice with 10 mM Tris-HCl, pH 7.4, 150 mM NaCl, 0,25% Nonidet P-40, once with 5mM Tris-HCl, and eluted with sample buffer before 10% SDS-Polyacrylamide gel electrophoresis.

**Mass spectrometry**

To identify the proteins in the immunoprecipitated, the gels were divided into 10 pieces, and each underwent trypsin in gel digestion procedure. NanoUPLC-hrMS/MS analyses of the resulting peptides mixtures were carried out on a Q-Exactive orbitrap mass spectrometer (Thermo Fisher Scientific), coupled with a nanoUltimate300 UHPLC system (Thermo Fisher Scientific). Peptide separation was performed on a capillary BEH C18 column (0.075 mm X 100 mm, 1.7 µm, Waters) using aqueous 0.1% formic acid (A) and CH3CN containing 0.1% formic acid (B) as mobile phases and a linear gradient from 5% to 50% of B in 90 min and a 300 nL/min flow rate. Mass spectra were acquired over an m/z range from 400 to 1800. To achieve protein identification and relative quantization, MS and MS/MS data underwent Protein Discoverer (v4.1, Thermo Fisher Scientific) analysis using the non-redundant Data Bank UniprotKB/Swiss-Prot (Release 2021_03).

Proteins that were also identified in the cells transfected with the empty plasmid were considered artifacts and removed from the list. The others were compared for their relative abundance in the samples overexpressing LAV-BPIFB4.

**Experiments using vascular cells**

*Fluorescence cytochemistry assays*

Cells were seeded onto fibronectin-coated (1µg/mL) sterile glass coverslips. Semiconfluent slides were fixed with 4% buffered PFA at room temperature (RT). MitoSOX Red (Thermo Fisher Scientific) was employed to assess mitochondrial superoxides, following the manufacturer’s protocol. The lipofuscin content was determined as described previously.^5^ Supplementary Table 6 summarizes the conditions employed for immunofluorescence assays. Images were acquired employing either an epifluorescence (Leica DMI 6000B, Leica, Wetzlar, Germany) or a confocal (Leica TCS-SP8) microscope.

*Detection of senescent cells*

Double staining for Ki67 and *γ*H2AX was performed as described,^6^ using an epifluorescence microscope and conditions reported in **Supplementary Table 6.** Moreover, a fluorometric β-Galactosidase Detection Kit (Abcam) was used to measure the β-Galactosidase activity according to the manufacturer’s instructions. Briefly, cell lysates obtained from HUVECs at passage 3 and passage 10 were combined with FDG working solution and added to 96-well plates. Plates were incubated at 37 °C for 4 h. β-galactosidase activity was recorded by measuring the fluorescence intensity through a microplate reader (BioTeck, Synergy 2, Winooski, USA) at ex490/ex525 nm after the addition of the stop solution.

*In vitro angiogenesis assay*

The Matrigel assay (BD Biosciences, Franklin Lakes, USA) was performed on HUVECs supplemented with WT- BPIFB4 and LAV-BPIFB4 recombinant proteins (20 ng/ml) or vehicle for 24 h and on HUVECs and PCs in co-culture, where the latter had been (i) preconditioned with BPIFB4 recombinant proteins (20 ng/ml, for 24 h) or (ii) silenced for NCL before being preconditioned with BPIFB4 recombinant proteins, or (iii) transfected with a plasmid encoding the *LAV-BPIFB4* whole sequence or the sequence lacking the domains for the NCL interaction.

*Migration Scratch Assay*

Cells were plated at confluence in fibronectin-coated 24-well plate under the supplementation of recombinant BPIFB4 proteins and vehicle (20ng/ml) for 24 h. Then a scratch was produced in the center of each well using a p100 tip. Distance between the two edges was measured immediately after the creation of the scratch (D0) and after 7 h incubation (D1) with basal medium deprived of all growth factors but supplemented with 2% FBS. Percentage of gap closure (%GAP) was calculated as following: %GAP=100-(100*D1/D0).

*Cell Viability Assay*

The 3-(4,5-dimethylthiazol-2-yl)-2,5-diphenyltetrazolium bromide (MTT) assay was applied to evaluate cell viability. IHF-PCs and HUVECs were plated onto 96-well plates at a density of 5000 cells/well. After 24 hours, cells were conditioned with BPIFB4 recombinant proteins and vehicle (20ng/ml) for additional 24 h and subsequently exposed to MTT (1 mg/ml) (Invitrogen) for 4 hours at 37 °C. Formazan release was quantified at 570 nm using a Microplate Reader (Biotek).

*Human angiogenesis antibody array*

For the simultaneous detection of 43 angiogenesis-related proteins in IHF-PC conditioned medium, we used the Human Angiogenesis Antibody Array - Membrane (43 Targets) from Abcam (ab193655) (Cambridge, UK). Samples were collected as follows: IHF-PCs were cultured for 48 h in basal medium (ECBM2, PromoCell) deprived of all growth factors but supplemented with 2% FCS. After this time, cells were conditioned with the recombinant LAV-BPIFB4 protein or vehicle control, both at a final concentration of 20 ng/mL in basal ECBM2 medium without growth factors and FCS, for 24 h. After treatment, the medium was replaced with fresh basal ECBM2 medium for a further 24 h. This medium was finally collected, centrifuged at 5,000 *g* for 10 min and stored at -80 ^o^C until analysed.

The antibodies arrays were performed according to the manufacturer instructions, using 1 ml of PC-conditioned medium per membrane and incubating the samples with the membranes for 16 h at 4 ^o^C , with gentle shaking. Membranes were developed using a ChemiDoc MP Imaging System (Bio-Rad). Images were processed using the BioRad Image Lab software, and densitometry analyses were carried out using the ImageJ software. Briefly, the background was subtracted from each target and the average signal density of the two spots was calculated. Afterwards, average signals on membranes were normalised to 6 positive control spots intensities to allow the comparison across different membranes, as recommended by the vendor. Finally, relative protein expression levels in the LAV group were determined as a fold-change against the respective vehicle group.

**Gene therapy in middle-aged and older mice**

Experimental procedures were compliant with the EU Directive 2010/63/EU and principles stated in the Guide for the Care and Use of Laboratory Animals (Institute of Laboratory Animal Resources, 1996). The protocols detailed below were prepared with support from the Experimental Design Assistant, a free resource from the National Centre for Replacement, Refinement, and Reduction of Animals in Research (https://eda.nc3rs.org.uk/). Mice were housed in groups of 1–6 animals (as required by the experimental procedure) in an enriched environment within a bio-secure unit under a 12 h light/dark cycle, fed with EURodent Diet (5LF5, LabDiet, Durham, UK) and given drinking water *ad libitum*. The GraphPad software (https://www.graphpad.com/quickcalcs/randomize1/) was used to randomly assign subjects to treatment groups, which were coded to allow a blind assessment of the data.

*Objective:* The studies, conducted at the University of Bristol (U.K.), aimed to assess the efficacy of *LAV-BPIFB4* gene therapy in halting cardiac dysfunction caused by aging. A secondary objective was to determine if the efficacy was affected by the sex and age of the experimental subjects.

*Protocol:* The experimental procedures were approved by the British Home Office (PPL 30/3373). One week after baseline echocardiography, 14-month old (*early study:* male and female) or 18-month old (*late study:* female) C57Bl/6J mice (Charles River) were randomized to receive an AAV-vector (100 μL of a master solution containing 1 × 10^12^ GC/mL) or an equivalent volume of vehicle (PBS) (ratio of sample size = 3:1) through the tail vein, with the mice under isoflurane anesthesia (2-3%). The AAV arm comprised three subgroup treatments: *AAV9-LAV-BPIFB4*, *AAV9-WT-BPIFB4*, *AAV9-GFP* (ratio of sample size = 1:1:1). Mice were examined weekly for 4 months (*early study*) or 1 month (*late study*), with a final echocardiography examination performed at the end of this period. Animals were terminated under isoflurane anaesthesia by exsanguination followed by removal of tissues and organs for histology and molecular biology. Subgroups of mice from *early* and *late* *studies* were transferred using a certified transporter to the Wales Research and Diagnostic Positron Emission Tomography Centre (PETIC) in Cardiff (UK). After one-week of adaptation to the environment, animals underwent an assessment of basal and stress myocardial perfusion using cardiac positron emission tomography/computed tomography (PET/CT) imaging. *Endpoints:* Cardiac index (primary endpoint) and vascular density (secondary endpoint).

*Null hypothesis:* The changes in cardiac index from baseline to final measurement and capillary density are not affected by experimental manipulation (*AAV9-LAV-BPIFB4*) to be tested. Therefore, there is no difference in the outcome measures as compared with the control (vehicle) or the other *AAV9* groups, using posthoc pairwise comparisons after ANOVA.

- *Echocardiography*

Dimensional and functional parameters were measured in mice of the *early study* (n=10 females and n=3 males/treatment group) and *late study* (n=10 females/treatment group) by an investigator blind to the randomization protocol using a Vevo3100 echocardiography system (VisualSonics).^7^ The analyses were performed with mice under isoflurane anesthesia (2.5% for induction, followed by 0.5–3.0% as appropriate to maintain heart rate close to 450 bpm).

- *Micro-PET/CT perfusion*

The myocardial perfusion was measured in the Bristol study on older mice but not in the Rouen study on MI mice, as the PET/CT imaging was only available in Bristol. The PET/CT scans were performed with mice under isoflurane anaesthesia (3-5% isoflurane in O_2_ for induction and 1.5-2% for maintenance). Intravenous (IV) and intraperitoneal (IP) routes were used for the administration of radiopharmaceutical and pharmacological stress agents, respectively, following adaptation of published prtocols.^8, 9^

Each mouse had a 10 min ammonia rest scan, a 10 min ammonia stress scan, and a CT scan. Briefly, ^13^N-ammonia (30-100 MBq in 20-60 µLi) was infused over 10 sec interval through an IV cannula using an infusion pump. The actual acquisition started a few seconds before the administration of ^13^N-ammonia to capture an infusion pump infused 13N-ammonia (30-100 MBq in 20-60 µLi) over 10 sec interval through an IV cannula tion of dobutamine (2.5 μg/kg in 50 µL),^9^ allowing a 5 min period for a cardiac stress event to occur before initiating the stress scan. When the heart rate increased to about 600 bpm (measured by ECG pads placed on paws), a second dose of ^13^N-ammonia (30-100 MBq IV in 20-60 µL) was infused over 10 sec. The image acquisition was also initiated a few seconds before the radiotracer injection by applying the same image acquisition process as in the rest scan. Finally, a whole-body CT scan with a duration of 5 min (480 projections, 2 rotations, 50keV) was performed to calculate attenuation corrections as well as allowing the co-registration of images which in turn gives a better visualization of the structural and functional information from the CT and PET, respectively.

All PET scans were acquired in list mode acquisition and images were reconstructed with the same voxel size of 400 µM using interview fusion software. The framesets of both rest and stress scans were as follows: 35 frames (12x5 sec; 18x30 sec, with a whole duration of 10 min). CT scans were reconstructed with a 250µM voxel size and Sinograms were produced using the 2-dimentional filtered back-projection method. CT-based attenuation and scatter corrections, as well as normalization, were applied to the reconstructed images. Following the reconstruction, all images were exported to PMOD software for further Kinetic modelling and data analysis.

**Tissue collection and histological analysis**

Blood was obtained into EDTA directly from the heart through a 23 gauge cannula. The heart was then flushed with 1% EDTA in normal phosphate-buffered saline (PBS), weighed, and then cut into pieces; the top 2/3 section was drop-fixed in 4% PFA, and the lower third was separated into the right ventricle (RV) and left ventricle LV and flash frozen. All PFA samples were kept at +4^o^C for 18-24 h and then the PFA was replaced with PBS. The PFA-fixed cardiac tissues were cryoprotected using 30% sucrose for 24 h before being embedded in OCT. Histochemical and immunohistochemical studies were performed on 4 μm thick sections cut using a Thermo Fisher Scientific CryoStar NK50 cryostat unless otherwise stated.

**Histological analyses of mouse hearts**

Specific antibodies and procedures are listed in **Supplementary Table 7**. All the immunochemical procedures included tissue sections without primary antibodies as a technical internal control and suitable irrelevant IgG as negative controls. Images were acquired employing either a transmitted light microscope (Leica DMD 108, Leica, Wetzlar, Germany or Olympus BS40), a confocal microscope (Leica TCS-SP8), or an epifluorescence microscope (Leica DMI 6000B or Zeiss AxioObserver.Z1 microscope).

Cryosections were stained using hematoxylin and eosin (H&E) or elastic van Gieson (EVG) protocols using a Shandon Varistan 24-4 slide stainer (Thermo Fisher Scientific). Slides were mounted with DPX. Further 8-μm thick sections were stained for collagen using the Azan Mallory method (Heidenhain’s adaption of Mallory’s trichrome stain). A senescence β-galactosidase staining kit (Cell Signalling Technology #9860) was used to identify senescent cells in the frozen cardiac sections (20-30 fields, x400 magnification). In addition, senescent cardiomyocytes and interstitial cells were recognized using anti-mouse P16ink4A and expressed as the number of positive nuclei per mm^2^ of tissue. Cardiac muscles were stained with anti-α-sarcomeric actin (1:200, mouse IgM isotype, Sigma-Aldrich), for 2 hours, at RT and Alexa Fluor 647-conjugated anti-mouse IgM (1:200, for 1 hour, at +20°C, Life Technologies, UK) was used as a secondary antibody. Slides were stained with 1:1000 (v/v) DAPI solution in 1xPBS and mounted with Fluoromount G for the imaging. Representative and quantitative images were taken using a Zeiss Observer.Z1 microscope set up on a fluorescent field light path with a 20x objective. The P16ink4A positive cells were expressed as the number of positive nuclei per mm^2^ of tissue using Image J software (http://rsbweb.nih.gov/ij). Cardiac samples were assessed for apoptosis using a TUNEL assay according to the manufacturer’s instructions (ApopTag® Red In Situ Apoptosis Detection Kit, Millipore S7165).

Vascular density was measured by counting capillaries and arterioles in >10 fields (200 x magnification). Final data were expressed as the number of capillaries and arterioles per mm^2 7^. Morphometric analyses were carried out employing ImageJ software

Analysis of PCs associated with the coronary microvasculature was carried out on 2 to 3 sections/sample stained with antibodies anti-PDGFRβ (1:50, overnight +4°C, identifying PCs) and α-sarcomeric actin (1:100, overnight at +4°C), and with isolectin-B4 (IB4, 1:200, overnight +4°C, identifying vessel ECs). For analysis of PC density, 10 images with capillaries in cross-section were snapped using a 20x objective. The density of PCs was expressed as the number of IB4- PDGFRβ+ perivascular cells/mm^2^ of α-sarcomeric actin+ myocardial area. For the analysis of PC coverage, >10 images with capillaries longitudinally oriented were snapped using a 20x objective. The PC coverage was quantified as the ratio between the PDGFRβ and IB4 pixel counts, as previously reported.^10^ PCs surrounding large arteries, as well as epicardial PCs, were excluded from both analyses. Morphometric analyses were carried out employing ImageJ software.

Immunostaining for BPIFB4 was carried out on the PFA-fixed cardiac tissues cryoprotected using 30% w/v sucrose for 24 h before being embedded in OCT. Immunohistochemical studies were performed on frozen 8- μm-thick sections cut using the cryostat STAR Nx50 (Thermo Fisher, United Kingdom) set at −20°C. The sections were hydrated in 1xPBS for 10 min at RT and antigen retrieval was performed using citrate buffer 1x (Sigma-Aldrich, United Kingdom) PH=6, for 15 minutes, at +98 °C to unmask the epitope within the tissue. The blocking solution containing 5% v/v goat serum was used to stop the non-specific binding. Antibody staining was performed using an anti-α-sarcomeric actin (1:200, mouse IgM isotype, Sigma-Aldrich, United Kingdom), for 2 h, at RT to recognise cardiac muscle, and Alexa Fluor 647-conjugated anti-mouse IgM (1:200, for 1 hour, at +20°C, Life Technologies, United Kingdom) was used as a secondary antibody. A rabbit anti-BPIFB4 (GeneTex, United Kingdom) was used as primary antibody at 1:100, and biotinylated Isolectin GS-IB4 (1:200, Life Technologies, United Kingdom) was employed to stain the capillaries. Alexa Fluor 568 conjugated anti-rabbit and Alexa Fluor Streptavidin 488 were used 1:200 for BPIFB4 and IB4, respectively. Nuclei were labelled with DAPI (1:1000, Life Technologies, United Kingdom) and slides were covered with aqueous mounting media (Fluoromount G, Life technologies, United Kingdom) and coverslips for fluorescent imaging. Images were taken using the Leica SP8 AOBS confocal laser scanning microscope attached to a Leica DM I8 inverted epifluorescence microscope and processed using LASX software. ‘Lightning’ adaptive image restoration was used to enable optimisation of resolution. Image J Fiji was used to merge the fluorescent-coloured channels and to add the scale bars.

**Statistical analyses**

In the *in vitro* and *in vivo* studies, the comparison among groups with one independent variable was performed using a Student’s *t*-test or with the equivalent non-parametric test following the assessment of normal distribution using Kolmogorov-Smirnov test or Shapiro-Wilk test. When appropriate, one-way ANOVAs (followed by Tukey’s multiple comparisons tests) or Kruskal-Wallis tests (followed by Dunn’s multiple comparison tests) were employed. Comparison among groups with 2 independent variables was performed employing repeated measurements two-way ANOVA followed by Sidak’s multiple comparison test. The significance level has been set to α = 0.05. Statistical analyses have been performed by the R software environment for statistical computing and graphics version 4.0.5 (www.r-project.org) except when specified.

**Supplementary Figure 1. Comparison of failing hearts from homozygous LAV and heterozygous or homozygous WT genotypes.**  **(A)** Similar expression of BPIFB4 in endothelial cells (Mann Whitney test) and myocytes (Unpaired t-test) from the two groups **(B)** Similar capillary and **(C)** arteriole density in the two groups (Unpaired t-test). **(D)** Higher PC coverage and density in LAV homozygous hearts (Unpaired t-test).

**Supplementary Figure 2. Increased expression of senescence markers in IHF hearts.** **(A)** Panels show examples of human C-PCs homogeneously expressing the typical markers NG2 (green), Platelet-Derived Growth Factor Receptor β (PDGFRβ) (red), TBX18 (green), and Nestin (red), in the absence of the fibroblast marker PDGFRα (green). **(B-E)** Compared with controls, IHF-PCs are more frequently marked as Ki67^neg^γ and H2AX^pos^ senescent cells (Ki67 stained green and γH2AX red) (**B**, n=10 C-PCs and 7 IHF-PCs), accumulate more oxidized lipofuscins (green) expressed as immunofluorescence intensity units (IFU) in their cytoplasm (**C**, n=6 C-PCs and 5 IHF-PCs), show increased mitochondrial superoxide content (red) (**D**, n=4 C-PCs and 4 IHF-PCs), and have reduced levels of the nuclear Vitamin D receptor (red) (**E**, n=5 C-PCs and 5 IHF-PCs). **(F-G)** Expression of BPIFB4 in C-PCs and IHF-PC (n=6 per group for PCR, and n=6 C-PCs and 5 IHF-PCs for fluorescence studies). Both one-step RT-PCR (left panel) and real-time-PCR (histograms in the right panel) showed that the BPIFB4 transcript is more abundant in C-PCs than IHF-PCs lines **(F)**. Immunofluorescence analysis confirmed lower BPIFB4 protein levels in IHF-PCs **(G)**. Data were analyzed using unpaired t-test (**panels B** and **D**) or the Mann-Whitney U test (all the other panels).

**
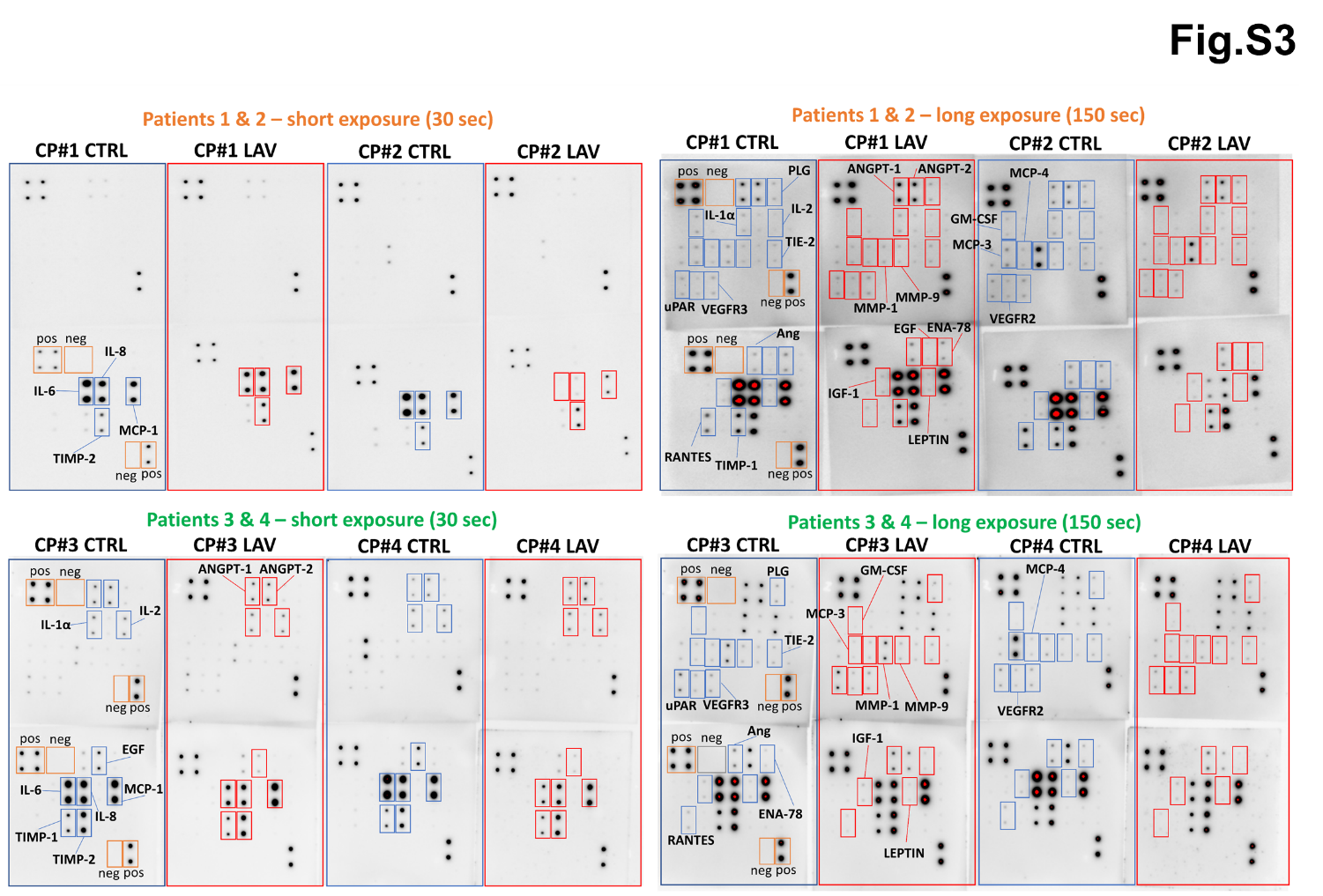
**

**Supplementary Figure 3.** Human angiogenesis antibody arrays. Qualitative analysis of angiogenesis-related factors released by human aged IHF-PC in the culture medium after conditioning with the recombinant LAV-BPIFB4 protein or vehicle (CTRL). N=4 patients’ cells. Each set of membranes was exposed for a shorter and a longer time as indicated, to allow for detection of proteins with different expression levels. Targets names are reported on the membranes. Pos = positive control spots. Neg = negative control. Red spots on the membranes with long exposure time indicate signal saturation of most abundant factors. Quantification of this factor was performed after the shorter exposure

**Supplementary Figure 4. Effect of LAV protein on HUVECs. (A)** Bar graph showing LAV-BPIFB4 did not affect the viability of HUVECs (early and late passages), as evaluated by MTT assay (n=5 per group). **(B)** HUVEC migration was assessed in a scratch assay. Representative phase-contrast images and a bar graph show that LAV-BPIFB4 supplementation promoted migration of late passage HUVEC. Images were acquired immediately after scratching and at 7 h thereafter. Dashed lines indicate the front of migration. N=5 per group. Data were analyzed using Ordinary one-way ANOVA.

d

**
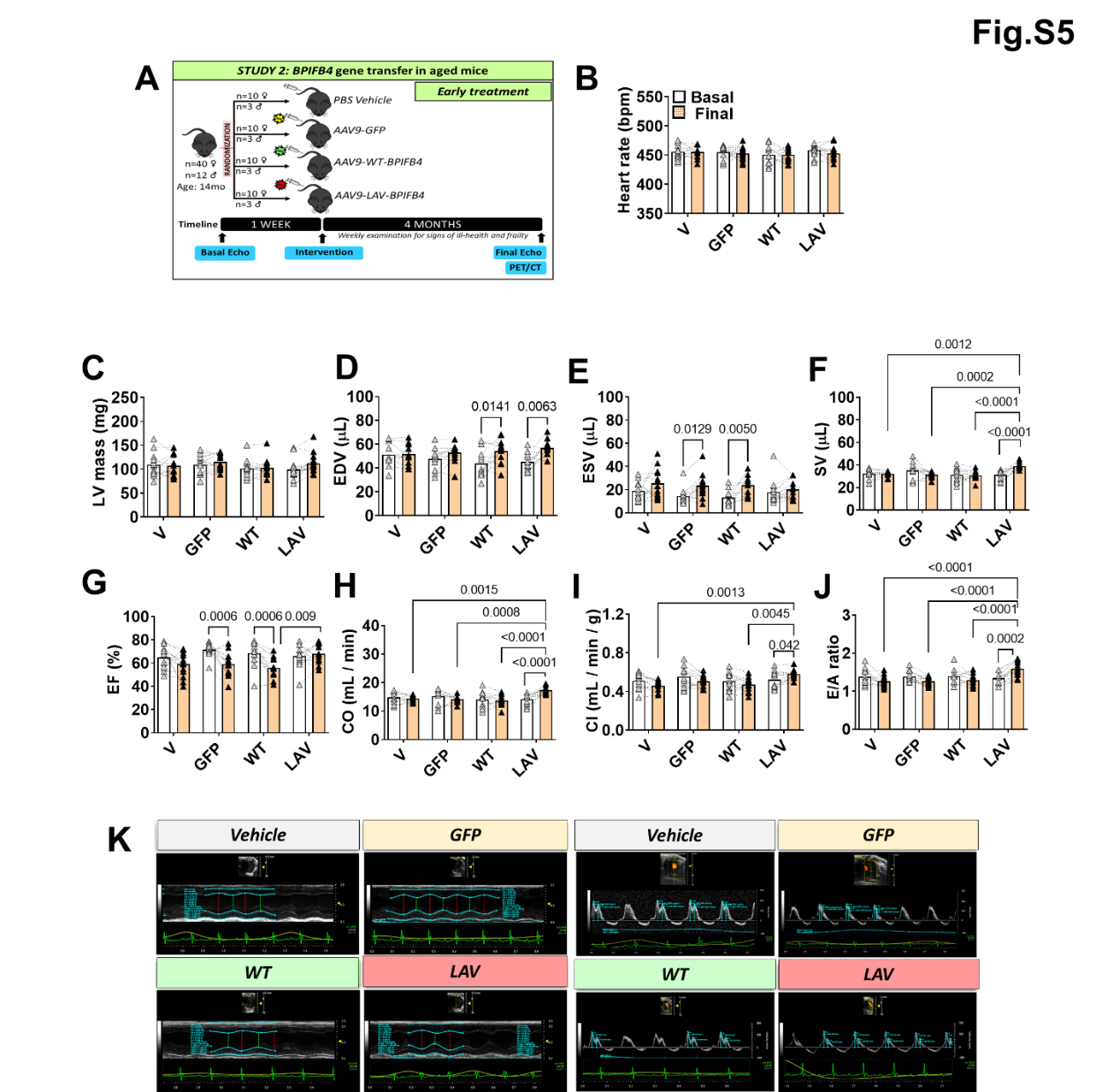
Supplementary Figure 5. A single systemic injection of *AAV-LAV* attenuates heart impairment in aging mice of the early study. (A)** Schematic of the protocol. **(B-J)** Baseline and final echocardiography data. Heart rate assessed using short-axis view **(B)**, left ventricular mass **(C)**, end-diastolic volume (EDV) **(D)**, end-systolic volume (ESV) **(E)**, stroke volume (SV) **(F)**, ejection fraction (EF) **(G)**, cardiac output (CO) **(H)**, cardiac index (CI) **(I)**, and E/A ratio **(J)**. Bar graphs show combined data for male and female mice, including the mean, standard deviation, and individual values. Statistical analysis was performed using 2-way ANOVA followed by Šídák's multiple comparisons test. **(K)** Representative echocardiography images.

**Supplementary Figure 6**. **Expression of BPIFB4 in the heart of mice given the vehicle or *AAV-LAV-BPIFB4*. (A-B)** Representative fluorescence confocal images showing areas of transversally sectioned myocardium. Vessels are identified by Isolectin B4 staining (green colour) and BPIFB4 (red pseudo-colour), cardiomyocytes are identified by α-sarcomeric actin staining (white pseudo-colour), and nuclei by DAPI staining (blue labelling). Objective: 63x; Scale bars: 20 μm.


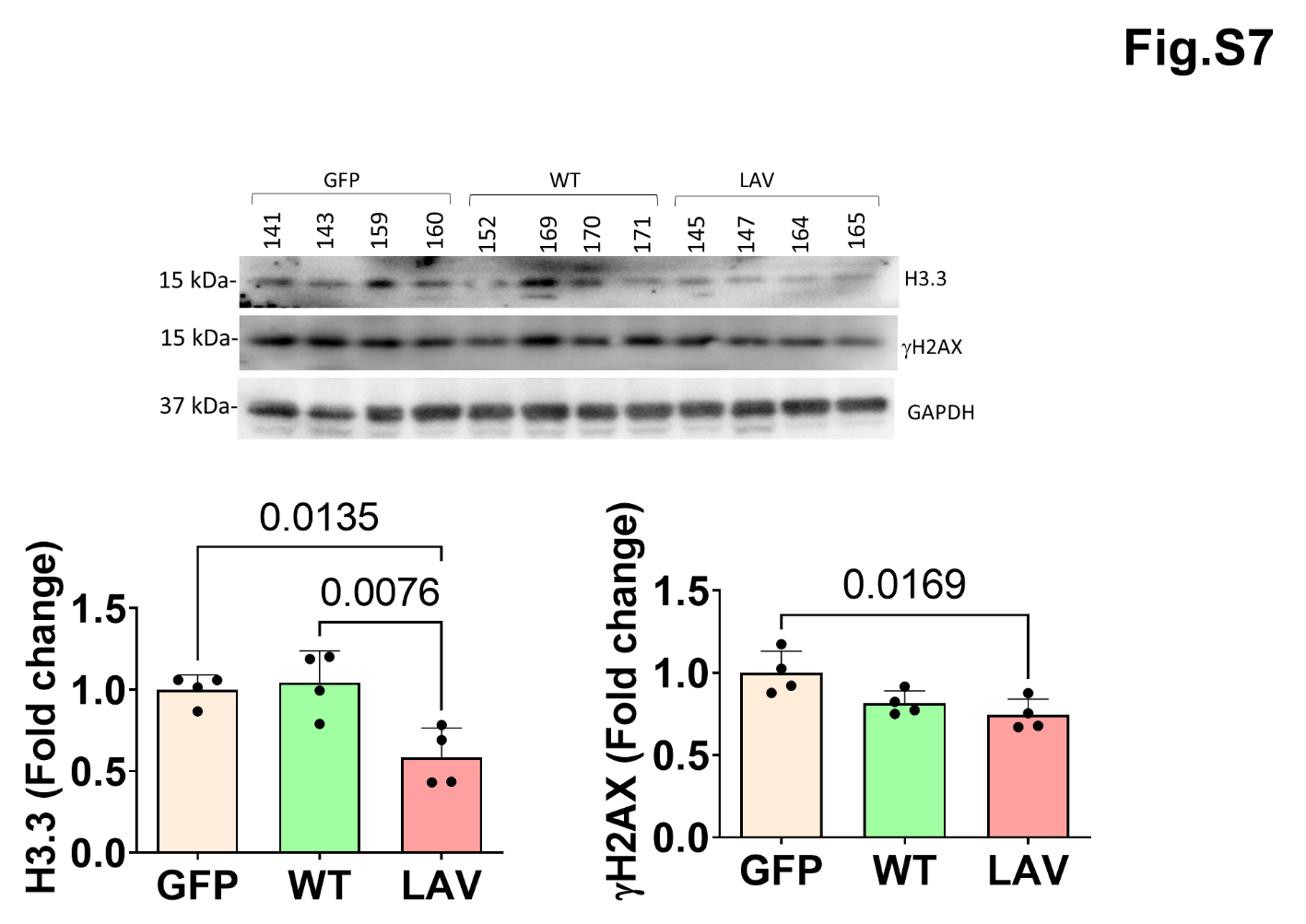


**Supplementary Figure 7**. ***AAV-BPIFB4 -LAV* attenuates cardiac cell senescence.** Western blot was performed on left ventricle samples from the early study. The histograms indicate expression values normalized relative to housekeeping signals. Data were expressed as fold changes relative to control (GFP). Data were analyzed using one-way ANOVA.

**
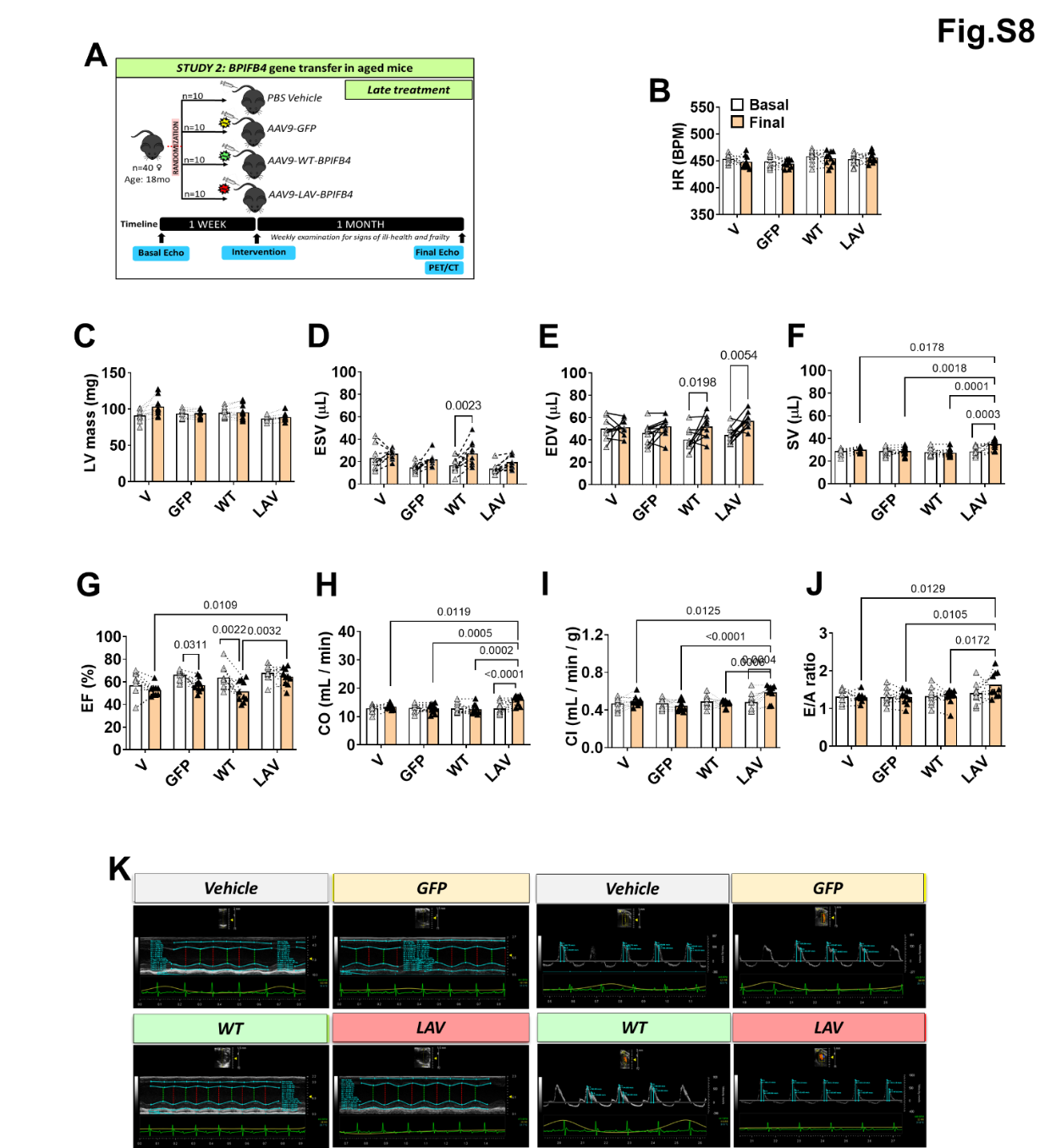
Supplementary Figure 8. Late *AAV-LAV-BPIFB4* gene therapy improves cardiac function in elderly mice. (A)** Schematic of the protocol. **(B-J)** Baseline and final echocardiography data. Heart rate (HR) assessed using short-axis view **(B)**, left ventricular mass **(C)**, end-diastolic volume (EDV) **(D)**, end-systolic volume (ESV) **(E)**, stroke volume (SV) **(F)**, ejection fraction (EF) **(G)**, cardiac output (CO) **(H)**, cardiac index (CI) **(I)**, and E/A ratio **(J)**. Bar graphs show combined data for male and female mice, including the mean, standard deviation, and individual values. Statistical analysis was performed using two-way ANOVA followed by Šídák's multiple comparisons test. **(K)** Representative echocardiography images.

**
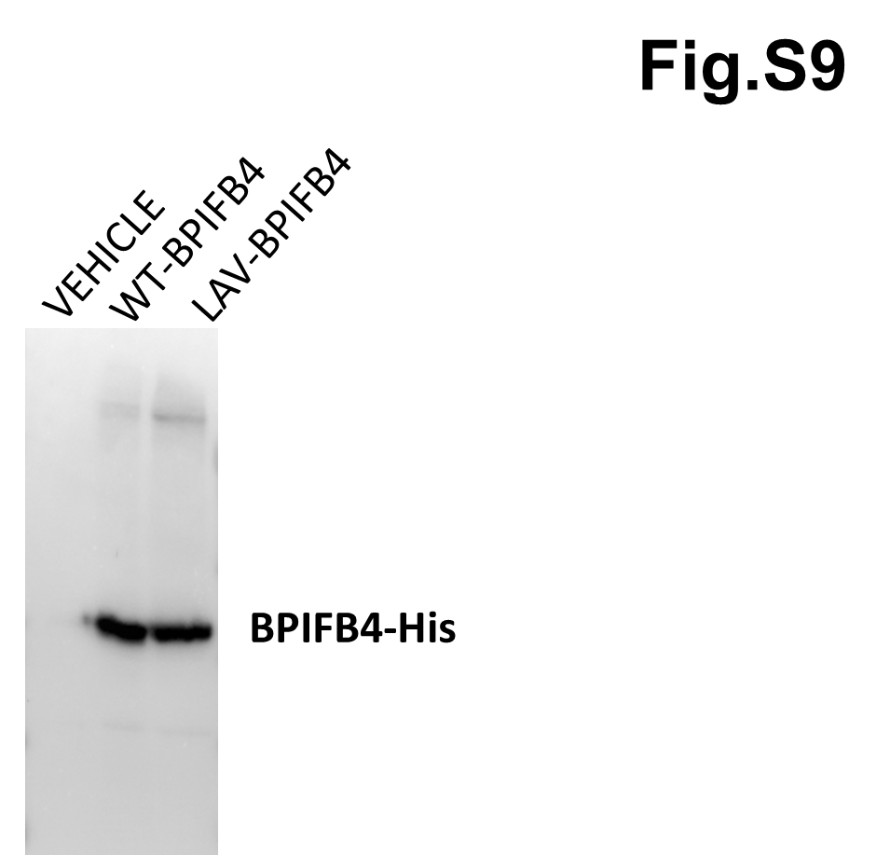
**

**Supplementary Figure 9. Quality control of purified recombinant proteins.** Aliquots of the protein samples purified from Hek-293 transfected with empty (vehicle) and *WT-* and *LAV-BPIFB4* vectors were separated by electrophoresis using 4–12% NuPAGE Bis-Tris protein gels and probed with antibody anti-BPIFB4. The image shows the specificity and purity of the recombinant proteins.

**Supplementary Table 1. Clinical and demographic data of elderly patients with IHF and control subjects whose hearts were employed for immunohistochemical analyses**

|  | **IHF** | | | | **Controls** | | | |  |
| --- | --- | --- | --- | --- | --- | --- | --- | --- | --- |
|  | **Obs** | **F-Miss (%)** | **Value** | **Distribution** | **Obs** | **F-Miss (%)** | **Value** | **Distribution** | ***p-value*** |
| **Age (years)** | 24 | 0 (0%) |  | 62.1±5.6 | 9 | 0 (0%) |  | 45.7±13.3 | <0.0001 |
| **Sex (%)** | 24 | 0 (0%) | M  F | 22 (92%)  2 (8%) | 9 | 0 (0%) | M  F | 3 (33%)  6 (67%) | 0.0017 |
| **Genotype** | 24 | 0 (0%) | LAV  Other | 7 (29%)  17 (71%) |  |  |  |  |  |
| **Time from MI (Months)** | 24 | 3 (12.5%) |  | 139.6±101.8 |  |  |  | N.A. | - |
| **Heart structural data** | | | | | | | | | |
| **LV Ejection Fraction (%)** | 24 | 9 (37.5%) |  | 24.3±3.9 | 9 | 5 (55%) |  | 58.7±6.3 |  |
| **Heart Weight (g)** | 24 | 1 (4%) |  | 498±94 | 9 | 3 (33%) |  | 405±154 |  |
| **Transverse Diameter (mm)** | 24 | 1 (4%) |  | 130±12 | 9 | 4 (44%) |  | 98±15 |  |
| **Inner Longitudinal Diameter (mm)** | 24 | 1 (4%) |  | 93±13 | 9 | 4 (44%) |  | 74±9 |  |
| **Wall Thickness (mm)** | 24 | 2 (8%) |  |  | 9 | 5 (55%) |  |  |  |
| **LV Ant** |  |  |  | 10±4 |  |  |  | 14±0 |  |
| **LV Lat** |  |  |  | 11±4 |  |  |  | 14±0 |  |
| **LV Post** |  |  |  | 10±3 |  |  |  | 14±0 |  |
| **RV** |  |  |  | 6±2 |  |  |  | 3±1 |  |
| **Septum** |  |  |  | 10±3 |  |  |  | 13±2 |  |
| **Risk factors** | | | | | | | | | |
| **Smoke History** | 24 | 1 (4%) |  | 52.1% |  |  |  | N.A. | - |
| **Hypertension** | 24 | 1 (4%) |  | 39.1% | 17 |  |  | N.A. | - |
| **Dyslipidemia** | 24 | 0 (0%) |  | 62.5% | 17 |  |  | N.A. | - |
| **Diabetes** | 24 | 1 (4%) |  | 26.1% | 17 |  |  | N.A. | -. |

N.A. Categorical variables distribution are described as absolute and relative frequency (%), numeric variables distribution is described as mean ± standard deviation. p-value = p-value from Mann-Whitney and Fisher’s exact test.

**Supplementary Table 2. Antibodies and experimental conditions employed for** **Immunohistochemical analyses on human hearts.**

| Antigen | Company (Catalog N°) | Dilution | Antigen retrieval | Incubation time and temperature | Secondary antibody | Incubation time |
| --- | --- | --- | --- | --- | --- | --- |
| BPIFB4 | GeneTex (GTX51455) | 1:300 | Citric buffer (pH6), 98°C, 40’ | O/N, 4°C | Alexa Fluor 555 1:800 | 1h, 37°C |
| CAV-1 | Biorbyt; orb213667 | 1:20 | Citric buffer (pH6), 98°C, 40’ | O/N, 4°C | Alexa4 Fluor 88 1:600 | 1h, 37°C |
| PDGFRβ | R&D Systems; AF385 | 1:40 | Citric buffer (pH6), 98°C, 40’ | O/N, 4°C | Alexa Fluor 647 1:600 | 1h, 37°C |
| α-Sarcomeric Actin | SIGMA  (A2172) | 1:400 | Citric buffer (pH6), 98°C, 40’ | 1h, 37°C | Cy5  1:400 | 1h, 37°C |
| Legend |  | | | | | |
| A488 | Alexa Fluor 488 labeled donkey Antibody | | | | | |
| A555 | Alexa Fluor 555 labeled donkey Antibody | | | | | |
| A633 | Alexa Fluor 633 labeled donkey Antibody | | | | | |
| Cy5 | Cy5 labeled donkey Antibody | | | | | |
| IHC | Envision Detection system Peroxidase/DAB (Agilent/DAKO), Rabbit/Mouse | | | | | |

**Supplementary Table 3. Demographic data of patients with ischemic heart failure (IHF) and control subjects** **whose hearts were employed to isolate pericytes**

|  | **IHF** | **Controls** |
| --- | --- | --- |
| **Number** | 14 | 15 |
| **Age (years)** | 58.1.0±7.3 | 50.6±11.4 |
| **Sex (M/F)** | 14/0 | 9/6 |

**Supplementary Table 4: Primers employed in Realtime RT-PCR experiments and cloning.**

| ***GENE*** | ***Forward*** | ***Reverse*** | | ***Application*** | |  |
| --- | --- | --- | --- | --- | --- | --- |
| ***BPIFB4***  ***β-actin***  ***BPIFB4*** | GGTCCTCAGGGTGACGAAAG  GAGACCGCGTCCGCC  GTGGGTGTCTACCTGAGCTTGT | | TATCACCAACACCCAAGGGC  ATCATCCATGGTGAGCTGGC  GCTCAATGACCAGCCGAGGATA | | RT-PCR  RT-PCR  Real-time PCR | |
| ***BPIFB4***  ***47S***  ***GAPDH***  ***BPIFB4^a^***  ***BPIFB4^B^*** | GGATATCACCAATGGCATGTT  CCTGCTGTTCTCTCGCGCGTCCGAG  AGGTGAAGGTCGGAGTCAAC  ACGAGACAAACACGGTCCTC  GAGGCGATCGCATGCTGCAGCAAAGTGATG | ATCAGGGCTCCCAGTGTG  AACGCCTGACACGCACGGCACGGAG  CCATGTAGTTGAGGTCAATGAAG  TGCGCTCAGCACCAAAAGGTC  GCGACGCGTTGCGCTCAGCACCAAAAG | | Real-time PCR for detection of deleted isoforms  Real-time PCR  Real-time PCR  Cloning in pGEM-T easy system  Cloning in pCMV6-Entry vector | |  |

**Supplementary Table 5. Antibodies and experimental conditions employed for Western blot.**

| Antigen | Company (Catalog N°) | Dilution |  | Host and  Clonality | Incubation time and temperature | Secondary antibody | Incubation time |
| --- | --- | --- | --- | --- | --- | --- | --- |
| BPIFB4 | Clinsciences  (Custom made) | 1:1000 |  | Rabbit,  Polyclonal | 16h, 4°C | ECL Rabbit Igg HPR linked 1:3000 | 1h, 4°C |
| NCL | Abcam; AB22758 | 1:1000 |  | Rabbit,  Polyclonal | 16h, 4°C | ECL Rabbit Igg HPR linked 1:3000 | 1h, 4°C |
| H3.3 | Abcam; AB176840 | 1:400 |  | Rabbit  Monoclonal | 16h, 4°C | ECL Rabbit Igg HPR linked 1:3000 | 1h, 4°C |
| ϒH2AX | millipore  05-636 | 1:500 |  | Mouse  Monoclonal | 16h, 4°C | ECL Mouse Igg HPR linked 1:3000 | 1h, 4°C |
| GAPDH | Sigma  G8795 | 1:3000 |  | Mouse  Monoclonal | 16h, 4°C | ECL Mouse Igg HPR linked 1:3000 | 1h, 4°C |
| Vinculin | Sigma  V9131 | 1:3000 |  | Mouse  Monoclonal | 16h, 4°C | ECL Mouse Igg HPR linked 1:3000 | 1h, 4°C |
| βeta-actin | Abcam;  AB6276 | 1:3000 |  | Mouse  Monoclonal | 16h, 4°C | ECL Mouse Igg HPR linked 1:3000 | 1h, 4°C |

**Supplementary Table 6: Antibodies and experimental conditions employed for cytochemistry analyses.**

| Antigen | Company  (Catalog N°) | Dilution | Antigen retrieval | Incubation time | Incubation temperature | Secondary antibody |
| --- | --- | --- | --- | --- | --- | --- |
| PDGFR-α | ThermoFisher  (MA5-38592) | 1:100 | Triton X, 1:100 | 2h | 37°C | A488 anti Mouse |
| pdgfr-β | ThermoFisher  (MA514851) | 1:100 | Triton X, 1:100 | O/N | 4°C | A555 anti Rabbit |
| tbx18 | ThermoFisher  (PA538563) | 1:100 | Triton X, 1:100 | O/N | 4°C | A555 anti Rabbit |
| NG2 | ThermoFisher  (37-2700) | 1:100 | Triton X, 1:100 | O/N | 4°C | A488 anti Mouse |
| Ki67 | Abcam (ab15580) | 1:800 | Triton X, 1:100 | 1h | 37°C | A488 anti Rabbit |
| γH2A.X | Merck (05-636) | 1:600 | Triton X, 1:100 | 1h | 37°C | A555 anti Mouse |
| Vitamin D Receptor | Novus Biological (NBP2-55786) | 1:50 | Triton X, 1:100 | O/N | 4°C | A488 anti Rabbit |
| Fibrillarin | Abcam (AB5821) | 1:100 | Triton X, 1:100 | 1h | 37°C | A488 anti Rabbit |
| BPIFB4 | Clinsciences  (Custom made) | 1:500 | Triton X, 1:1000 | 1h | 37°C | A488 anti Rabbit |
| Legend: |  |  |  |  |  |  |
| A488 | Alexa Fluor 488 labeled donkey Antibody | | | | | |
| A555  A647 | Alexa Fluor 555 labeled donkey Antibody  Alexa Fluor 647 labeled donkey Antibody | | | | | |

**Supplementary Table 7. Antibodies and experimental conditions employed for immunohistochemical analyses on mouse sections.**

| Antigen | Company (Catalog N°) | Dilution | Antigen retrieval | Incubation time and temperature | Secondary antibody | | Incubation time | |
| --- | --- | --- | --- | --- | --- | --- | --- | --- |
| α-Sarcomeric Actin | SIGMA (A2172) | 1:400  or 1:200 | Citric buffer (pH6), 98°C, 40’  or none | 1h, 37°C  or 2h, RT  or O/N, 4°C | Cy5 1:400  or Alexa Fluor 647 1:200  or TRITC 1:200 | 1h, 37°C  or 1h, RT | | |
| PDGFRβ | R&D Systems (AF1042) | 1:50 | none | O/N, 4°C | Alexa Fluor 647 1:200 | 1h, RT | | |
| Nucleolin | Abcam | 1:100 | Citric buffer (pH6), 98°C, 40’ | O/N, 4°C | Alexa Fluor 488 1:600 | 1h, 37°C | | |
| P16ink4A | Santa cruz biotechnology (sc-1661) | 1:50 | none | O/N, 4°C | Alexa Fluor 568 1:200 | 1h, RT | | |
| isolectin gs-Ib_4_-biotinILATED | Life Technologies (121414) | 1:200 | Citric buffer (pH6), 98°C, 30’  or none | O/N, 4°C | Streptavidin- Alexa Fluor 488 1:200 | 1h, RT | | |
| α-smooth muscle actin-Cy3 | Sigma C6198 | 1:400 | Citric buffer (pH6), 98°C, 30’ | O/N, 4°C | N/A | N/A | | |
| BPIFB4 | GeneTex (GTX51455) | 1:100 | Citric buffer (pH6), 98°C, 30’ | O/N, 4°C | Alexa Fluor 568 1:200 | 1h, RT | | |
| Legend: |  | | | | | | |  |
| A488 | Alexa Fluor 488 labeled donkey Antibody OR streptavadin | | | | | | |  |
| A555 | Alexa Fluor 555 labeled donkey Antibody | | | | | | |  |
| A568 | Alexa Fluor 568 labeled donkey OR goat Antibody | | | | | | |  |
| A633 | Alexa Fluor 633 labeled donkey Antibody | | | | | | |  |
| A647 | Alexa Fluor 647 labeled donkey OR goat Antibody | | | | | | |  |
| TRITC | TRITC labeled goat Antibody | | | | | | |  |
| Cy5 | Cy5 labeled donkey Antibody | | | | | | |  |
| IHC | Envision Detection system Peroxidase/DAB (Agilent/DAKO), Rabbit/Mouse | | | | | | |  |

**References**

1. Rolle IG, Crivellari I, Zanello A, Mazzega E, Dalla E, Bulfoni M, Avolio E, Battistella A, Lazzarino M, Cellot A, Cervellin C, Sponga S, Livi U, Finato N, Sinagra G, Aleksova A, Cesselli D, Beltrami AP. Heart failure impairs the mechanotransduction properties of human cardiac pericytes. *J Mol Cell Cardiol*. 2021; 151:15-30.
2. Avolio, E, Avolio E, Rodriguez-Arabaolaza I, Spencer HL, Riu F, Mangialardi G, Slater SC, Rowlinson J, Alvino VV, Idowu OO, Soyombo S, Oikawa A, Swim MM, Kong CH, Cheng H, Jia H, Ghorbel MT, Hancox JC, Orchard CH, Angelini G, Emanueli C, Caputo M, Madeddu P. Expansion and characterization of neonatal cardiac pericytes provides a novel cellular option for tissue engineering in congenital heart disease. *J Am Heart Assoc*. 2015; 4, e002043. doi: 10.1161/JAHA.115.002043.
3. Villa F, Carrizzo A, Spinelli CC, Ferrario A, Malovini A, Maciag A, Damato A, Auricchio A, Spinetti G, Sangalli E, Dang Z, Madonna M, Ambrosio M, Sitia L, Bigini P, Cali G, Schreiber S, Perls T, Fucile S, Mulas F, Nebel A, Bellazzi R, Madeddu P, Vecchione C, Puca AA. Genetic Analysis Reveals a Longevity-Associated Protein Modulating Endothelial Function and Angiogenesis. Circ Res. 2015;117:333-45.
4. Vecchione C, Villa F, Carrizzo A, Spinelli CC, Damato A, Ambrosio M, Ferrario A, Madonna M, Uccellatore A, Lupini S, Maciag A, Ryskalin L, Milanesi L, Frati G, Sciarretta S, Bellazzi R, Genovese S, Ceriello A, Auricchio A, Malovini A, Puca AA. A rare genetic variant of BPIFB4 predisposes to high blood pressure via impairment of nitric oxide signaling. *Sci Rep*. 2017; 7, 9706. doi: 10.1038/s41598-017-10341-x.
5. Gianfranceschi G, Caragnano A, Piazza S, Manini I, Ciani Y, Verardo R, Toffoletto B, Finato N, Livi U, Beltrami CA, Scoles G, Sinagra G, Aleksova A, Cesselli D, Beltrami AP. Critical role of lysosomes in the dysfunction of human Cardiac Stem Cells obtained from failing hearts. *Int J Cardiol*. 2016; 216, 140-50. doi: 10.1016/j.ijcard.2016.04.155.
6. Lawless C, Wang C, Jurk D, Merz A, Zglinicki Tv, Passos JF. Quantitative assessment of markers for cell senescence. *Exp Gerontol.* 2010; 45, 772-8. doi: 10.1016/j.exger.2010.01.018.
7. Dang Z, Avolio E, Thomas AC, Faulkner A, Beltrami AP, Cervellin C, Carrizzo A, Maciag A, Gu Y, Ciaglia E, Finato N, Damato A, Spinetti G, Alenzi A, Paisey SJ, Vecchione C, Puca AA, Madeddu P. Transfer of a human gene variant associated with exceptional longevity improves cardiac function in obese type 2 diabetic mice through induction of the SDF-1/CXCR4 signalling pathway. Eur J Heart Fail. 2020; 22:1568-1581.
8. Inubushi M, Jordan MC, Roos KP, Ross RS, Chatziioannou AF, Stout DB, Dahlbom M, Schelbert HR. Nitrogen-13 ammonia cardiac positron emission tomography in mice: effects of clonidine-induced changes in cardiac work on myocardial perfusion*. Eur J Nucl Med Mol Imaging*. 2004; 31, 110-6. doi: 10.1007/s00259-003-1328-5.
9. Calligaris, SD, Ricca M, Conget P. Cardiac stress test induced by dobutamine and monitored by cardiac catheterization in mice. *J Vis Exp.* 2013; 72**,** 50050. doi: 10.3791/50050.
10. Chintalgattu V, Rees ML, Culver JC, Goel A, Jiffar T, Zhang J, Dunner K Jr, Pati S, Bankson JA, Pasqualini R, Arap W, Bryan NS, Taegtmeyer H, Langley RR, Yao H, Kupferman ME, Entman ML, Dickinson ME, Khakoo AY.. Coronary microvascular pericytes are the cellular target of sunitinib malate-induced cardiotoxicity. *Sci Transl Med*. 2013; 5, 187ra69. doi: 10.1126/scitranslmed.3005066. PMID: 23720580; PMCID: PMC3833098.
